# Rectilinear Steiner Trees in Narrow Strips

**DOI:** 10.1007/s00454-026-00866-2

**Published:** 2026-08-05

**Authors:** Henk Alkema, Mark de Berg

**Affiliations:** Department of Mathematics and Computer Science, TU Eindhoven, Eindhoven, the Netherlands

**Keywords:** Computational geometry, Rectilinear Steiner trees, Fixed-parameter tractable algorithms, Approximation algorithms

## Abstract

A *rectilinear Steiner tree* for a set *P* of points in $\mathbb {R}^2$ is a tree that connects the points in *P* using horizontal and vertical line segments. The goal of Minimum Rectilinear Steiner Tree is to find a rectilinear Steiner tree with minimum total length. We investigate how the complexity of Minimum Rectilinear Steiner Tree for point sets *P* inside a δ-strip—that is, a strip of the form $(-\infty ,+\infty )\times [0,\delta ]$—depends on the strip width δ. We obtain three main results.

1. We present an algorithm that finds an optimal *monotonic* rectilinear Steiner tree in *O*(*n*) time, assuming the points are already sorted by *x*-coordinate. We show that when the difference between any two distinct *x*-coordinates is at least δ, then an optimal monotonic rectilinear Steiner tree for a point set inside a δ-strip is also optimal among all (possibly non-monotonic) rectilinear Steiner trees.
2. Next, we consider sparse points sets, that is, point sets such that any $1\times \delta$ rectangle inside the δ-strip contains *O*(1) points. We first show that an optimal monotonic rectilinear Steiner tree provides an O(δ)-approximation of an optimal rectilinear Steiner tree. Second, we present an algorithm with running time $n^{O(\sqrt{\delta })}$ that computes an optimal rectilinear Steiner tree for sparse point sets.
3. For point sets whose points are chosen randomly inside the rectangle $[0,n]\times [0,\delta ]$, we present an algorithm that is fixed-parameter tractable with respect to δ and linear in *n*. It has an expected running time of $2^{O(\delta \sqrt{\delta })} n$.

## Introduction

In the Minimum Steiner Tree problem in the plane, we are given as input a set *P* of points in the plane, called *terminals*, and the goal is to find a minimum-length tree that connects the terminals in *P*. Thus the given terminals must be nodes of the tree, but the tree may also use so-called *Steiner points* as nodes. Minimum Steiner Tree is a classic optimization problem. It was among the first problems to be proven NP-hard, not only for the case where the length of the tree is measured using Euclidean metric [13] but also in the rectilinear version [12]. It was also shown to be NP-hard for other metrics [6].

The rectilinear version of the problem, where the edges of the tree must be horizontal or vertical, is one of the most widely studied variants, and it is also the topic of our paper. The Minimum Rectilinear Steiner Tree problem dates back more than 50 years [14, 15]. Its popularity arises from its many applications, in particular in the design of integrated circuits [4, 5, 7, 22]. The two most important early insights on Minimum Rectilinear Steiner Tree came from Hanan [15] and Hwang [16]. Hanan observed that any terminal set *P* admits a minimum rectilinear Steiner tree (MRST, for short) whose edges lie on the grid formed by all horizontal and vertical lines passing through at least one terminal in *P*. This grid is often called the *Hanan grid*. This implies that the Minimum Rectilinear Steiner Tree problem can be reduced to a purely combinatorial problem—namely, a Steiner-tree problem on graphs—which is not possible for the Euclidean version of the problem. Hwang investigated the structure of optimal MRSTs in more detail, by providing a characterization of the different components of an MRST; see Sect. 2.

As mentioned, Minimum Rectilinear Steiner Tree can be considered a special case of the Steiner-tree problem on graphs. Here, the input is an edge-weighted graph G=(V,E) and a terminal set P⊆V, and the goal is to compute a minimum-length subtree of *G* that includes all terminals. In 1971 Dreyfus and Wagner [10] gave an algorithm solving the Steiner-Tree problem on graphs with positive integer weights in time $3^n \cdot \log W \cdot s^{O(1)}$, where *n* is the number of terminals, *s* is the total number of nodes, and *W* is the maximum edge weight in *G*. This was later improved by Björklund *et al.*  [3] and Nederlof [18], who gave an algorithm with $2^n \cdot W \cdot s^{O(1)}$ running time. A variant of the Dreyfus-Wagner algorithm for Minimum Rectilinear Steiner Tree runs in time $O(n^2 \cdot 3^n)$. Thobmorson *et al.*  [19] and Deneen *et al.*  [9] gave randomized algorithms for the special case of Minimum Rectilinear Steiner Tree where the terminals are drawn independently uniformly from a rectangle. Both algorithms run in $2^{O(\sqrt{n} \log n)}$ expected time. The question of whether an MRST can be found in time subexponential in the number of terminals for general (non-random) point sets was answered in 2018 by Fomin *et al.*  [11], who presented a $2^{O(\sqrt{n} \log n)}$ algorithm.

Due to the many applications of Minimum Steiner Tree variants in the plane, there has also been significant interest in practical implementations. These implementations rely on the insight that a minimum Steiner tree can always be decomposed into so-called *full components*, which are maximal subtrees that do not have any terminals as internal nodes [16]. (This holds for the Euclidean as well as the rectilinear version.) To compute an exact solution, a set of candidate full components is first computed and then it is computed which subset of candidate full components can be concatenated into an MRST. This process was introduced by Winter in 1985 [23], in his software package *GeoSteiner*. Still, only very small data sets could be handled, and even in 1994 the state-of-the-art software could solve the rectilinear variant of the problem for only up to 16 points [20]. Warme’s dissertation [21] significantly improved the process of concatenating the full components, resulting in optimal Steiner trees for up to 1,000 points for the rectilinear version of the problem and up to 2,000 points for the Euclidean version. In 1998 Althaus [2] obtained similar results. Throughout the years, GeoSteiner, which had become a collaboration between Warme, Winter and Zachariasen, has remained the fastest publicly available software package for computing minimum Steiner trees in the plane. By 2018, it could solve instances for up to 4,000 points for the rectilinear version, and up to 10,000 points for the Euclidean version [17].

**Our contribution.** The fastest known algorithm for Minimum Rectilinear Steiner Tree in $\mathbb {R}^2$ runs in $2^{O(\sqrt{n} \log n)}$ time [11]. In $\mathbb {R}^1$, on the other hand, the problem can be trivially solved in O(nlogn) time by just sorting the points. In order to better understand the computational complexity of the classic Minimum Rectilinear Steiner Tree problem in the plane, we therefore investigate how the complexity depends on the width of the terminal set *P*. If the point set in *P* is “almost 1-dimensional” in the sense that the points lie in a narrow strip $\mathbb {R}\times [0,\delta ]$, then can we solve Minimum Rectilinear Steiner Tree more efficiently than in the general case? And if so, how does the complexity scale with δ? Can we obtain an algorithm that is fixed-parameter tractable with respect to δ? This follows the line of research started recently by Alkema *et al.*  [1], who studied these questions for the Traveling Salesman Problem. We study these questions in the following three scenarios. Note that in all scenarios we assume that $P\subset \mathbb {R}\times [0,\delta ]$.

- *Minimum monotonic rectilinear Steiner trees.* A *monotonic* rectilinear Steiner trees is a rectilinear Steiner tree that intersects any vertical line in a connected set. We give a greedy algorithm that finds an optimal monotonic rectilinear Steiner tree in *O*(*n*) time, assuming the points are already sorted by *x*-coordinate. We then show that a minimum monotonic rectilinear Steiner tree (MMRST) is an MRST if the minimum difference between any two distinct *x*-coordinates of the points in *P* is at least δ; this threshold δ is tight in the worst case.
- *Sparse point sets.* We say that a point set *P* is *k-sparse* if for any $x \in \mathbb {R}$, the rectangle $[x,x+1]\times [0,\delta ]$ contains at most *k* points, for some sparsity parameter k⩾1. We first show that an MMRST for a *k*-sparse point set *P* provides an O(kδ)-approximation of an MRST for *P*. Next, we show that for *k*-sparse point sets in $\mathbb {R}^2$ an MRST must be *t*-tonic—an MRST is *t*-tonic if it intersects any vertical line at most *t*-times—for $t=O(k\sqrt{\delta })$, and we give a dynamic-programming algorithm that runs in $n^{O(k\sqrt{\delta })}$ time. We also show how to adapt this algorithm to a (1+ε)-approximation algorithm that runs in $(k/\varepsilon )^{O(k\sqrt{\delta }\log \delta )} n$ time.
- *Random point sets.* Our main result is for point sets *P* generated by taking *n* points independently uniformly inside a rectangle of height δ and width *n*. For this case we provide an FPT algorithm for Minimum Rectilinear Steiner Tree, which runs in expected time $2^{O(\delta \sqrt{\delta })}n$. More precisely, the expected running time is $\min \big ( n^{O(\sqrt{\delta })}, 2^{O(\delta \sqrt{\delta })}n \big )$. Note that the running time is linear when δ=O(1).

## Preliminaries

**Notation and terminology.** Let $$P:=\{p_1,\ldots ,p_n\}$$ be a set of *terminals* in a 2-dimensional horizontal strip with height δ —we call such a strip a δ*-strip*—which we assume without loss of generality to be $$\mathbb {R}\times [0,\delta ]$$. We use $$x_i$$ and $$y_i$$ to denote the *x*- and *y*-coordinate of point $$p_i$$, respectively. The points can be easily sorted on their *x*-coordinates: this can be done in O(nlogn) time for sparse point sets, and in *O*(*n*) expected time for random point sets [8]. Therefore, we will from now on assume that we are given the points ordered on their *x*-coordinate, i.e., $$x_i \leqslant x_j$$ for all $$1\leqslant i \leqslant j \leqslant n$$. We define the *spacing* of $$p_i$$ (in *P*) as $$\Delta _i :=x_{i+1} - x_i$$, for all $$1 \leqslant i \leqslant n-1$$. We write *P*[*i*, *j*] to denote the set $$\{p_i,\ldots ,p_j\}$$. We denote the vertical distance between two horizontal edges $e,e^{\prime }$—that is, the difference between their *y*-coordinates—by $$\textrm{dist}(e,e')$$. Similarly, for two vertical edges $e,e^{\prime }$ we use $$\textrm{dist}(e,e')$$ to denote their horizontal distance.

**Fig. 1:**
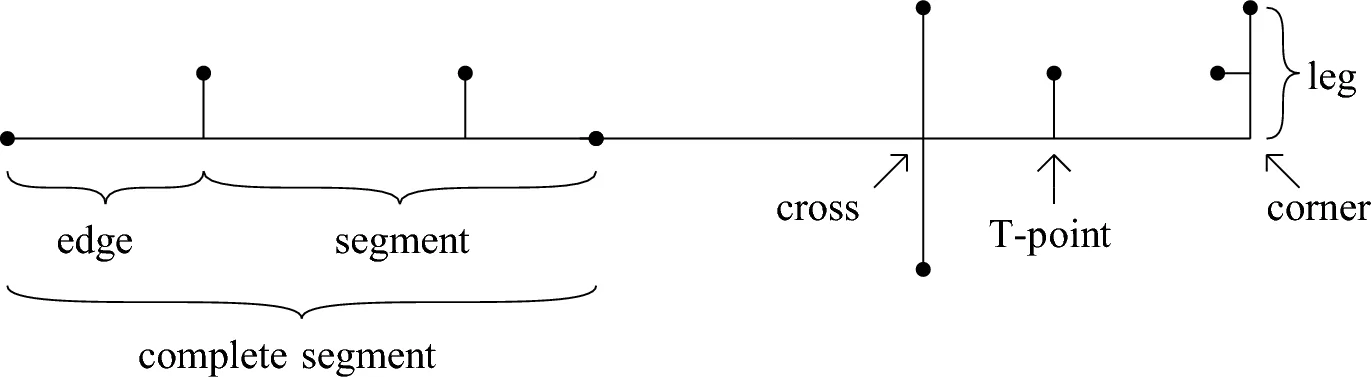
Illustration of terminology concerning rectilinear Steiner trees

Next we give some (mostly standard) terminology concerning rectilinear Steiner trees; see also Figure 1. A *rectilinear tree* is a tree structure embedded in the plane whose edges are horizontal or vertical line segments overlapping only at their endpoints. The *length* of a tree *T*, or $\Vert T\Vert$, is the sum of the lengths of its edges. A *rectilinear Steiner tree* for a set *P* of terminals is a rectilinear tree such that each terminal p∈P is an endpoint of an edge in the tree. A *minimum rectilinear Steiner tree (MRST)* is such a tree of minimum length.

The *degree* of a (Steiner or terminal) point *q* in a tree *T* is the number of edges incident on it. We denote the degree of *q* in *T* by ${{\,\textrm{degree}\,}}_T(q)$, or simply ${{\,\textrm{degree}\,}}(q)$ when *T* is clear from the context. Without loss of generality, if a degree-2 point has collinear (i.e. both horizontal or both vertical) incident edges then that point must be a terminal. Clearly, a point has degree at most 4. A point with degree of at least 3 that is not a terminal is called a *Steiner point*. A *corner* is a degree-2 point with non-collinear incident edges that is not a terminal. Hence, each endpoint of an edge is either a terminal, a Steiner point, or a corner.

A *segment* is defined to be a sequence of one or more adjacent collinear edges, with no terminals in the segment’s interior.^1^ A *complete segment* is a inclusion-wise maximal segment. Note that a complete segment does not have terminals in its interior. A corner is incident to exactly one horizontal complete segment and exactly one vertical complete segment. These complete segments are the *legs* of the corner. A *T-point* is a degree-3 Steiner point. Finally, a *cross* is a degree-4 Steiner point. Note that the endpoints of a complete segment are T-points, corners or terminals.

Separators will play a crucial role in our algorithms. A *separator* is a vertical line, not containing any of the points in *P*, that separates *P* into two non-empty subsets. For all 1⩽i<n such that $x_i < x_{i+1}$, we define $s_i$ to be the separator with *x*-coordinate $(x_i + x_{i+1})/2$. The *tonicity* of a rectilinear tree *T* *at a separator* *s* is the number of times *T* crosses *s*; when the tonicity of *T* at *s* is 1, we call it *monotonic* at *s*. The *tonicity* of a rectilinear tree *T* is the maximum over the tonicity of *T* at all separators. A rectilinear tree is called *monotonic* when its tonicity is 1.

**Characterisation of the MRST.** One of the most important properties of the Minimum Rectilinear Steiner Tree problem is the following:

### Observation 1

(Hanan [15]) Let *P* be a set of terminals in $\mathbb {R}^2$. Then there exists an MRST on *P* that is a subset of the *Hanan grid*, which is the grid formed by taking all horizontal and vertical lines that pass through at least one of the points of *P*.

From now on, we will only consider rectilinear Steiner trees that lie on the Hanan grid.

The next observation gives another useful property: parallel edges of an MRST cannot be too close to each other. Recall that $\Delta _i$ denotes the horizontal spacing between $p_i$ and $p_{i+1}$, and that δ denotes the height of the strip containing *P*. Also recall that $s_i$ is the separator in between the points $p_i$ and $p_{i+1}$.

### Observation 2

(i) Let $$E = \{e_1,\ldots ,e_m\}$$ be a set of *m* horizontal edges of an MRST *T* that all intersect two vertical lines ℓ and $\ell^{\prime }$. Then $$m \leqslant 1+\lfloor \delta / \textrm{dist}(\ell ,\ell ') \rfloor $$. A similar statement holds when *E* is a set of vertical edges intersecting two horizontal lines.
(ii) If $\Delta _i > \delta$, then the tonicity of any MRST at $s_i$ is 1.

### Proof

**Fig. 2:**
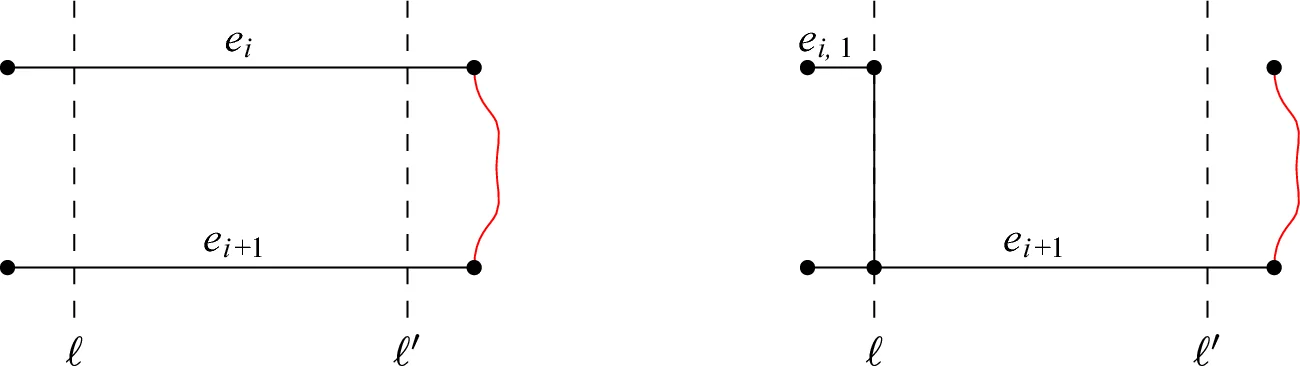
Illustration for the proof of Observation 2. On the left, *T*. On the right, $T^{\prime }$. Since the deleted part of $$e_i$$ is longer than the vertical edge connecting $$e_{i,1}$$ to $$e_{i+1}$$, the tree $T^{\prime }$ is shorter than *T*

We will first prove (i). Assume wlog that the edges in *E* are numbered from top to bottom, and let ℓ lie to the left of $\ell^{\prime }$. Suppose for a contradiction that $$m > 1 + \lfloor \delta /\textrm{dist}(\ell ,\ell ') \rfloor $$. Since $$\textrm{dist}(e_1,e_m) \leqslant \delta $$, there are two edges $$e_{i}$$ and $$e_{i+1}$$ such that $$\textrm{dist}(e_i,e_{i+1}) \leqslant \delta / (m-1) < \textrm{dist}(\ell ,\ell ')$$. We will now create a rectilinear Steiner tree $T^{\prime }$ strictly shorter than *T*, giving the desired contradiction. To this end we first delete the part of $$e_{i}$$ between ℓ and $\ell^{\prime }$. Let $$e_{i,1}$$ denote the part of $$e_i$$ to the left of ℓ (if any) and let $$e_{i,2}$$ denote the part of $$e_i$$ to the right of $\ell^{\prime }$ (if any); see Figure 2.

The deletion splits *T* into two components. Assume without loss of generality that $$e_{i,2}$$ is in the same component as $$e_{i+1}$$. By deleting $$e_{i,2}$$ and connecting $$e_{i,1}$$ to $$e_{i+1}$$ with a vertical edge contained in ℓ, we create a rectilinear Steiner Tree $T^{\prime }$ such that

1 $$\begin{aligned} \Vert T'\Vert = \Vert T\Vert - \textrm{dist}(\ell ,\ell ') - \Vert e_{i,2}\Vert + \textrm{dist}(e_i, e_{i+1}) < \Vert T\Vert , \end{aligned}$$

giving the desired contradiction.

Part (ii) of the observation directly follows from part (i). To see this, let $$\Delta _i > \delta $$ for some *i*. Then there are two lines ℓ and $\ell^{\prime }$ between $$p_i$$ and $$p_{i+1}$$ such that $$\textrm{dist}(\ell ,\ell ') > \delta $$. Let *T* be an MRST. Note that any edge of *T* that crosses $$s_i$$ also crosses ℓ and $\ell^{\prime }$. Therefore, by part (i) we know that *T* crosses $$s_i$$ at most $$m \leqslant 1 + \lfloor \delta /\textrm{dist}(\ell ,\ell ') \rfloor = 1$$ times. □

Next, we present a detailed characterization of the structure of an MRST, as given by Hwang [16]. To this end, define a *full component* of a rectilinear Steiner tree *T* to be a maximal subtree that does not have any terminals as internal nodes. Note that a node in a full component of an MRST is a terminal if and only if it is a leaf in that component. Also note that any terminal $p_i\in P$ will be a leaf in exactly $\text{ degree }(p_i)$ full components. Hwang’s theorem is now given by the following:

### Theorem 3

(Hwang [16]) Let *P* be a set of terminals in $\mathbb {R}^2$. Then there exists an MRST *T* on *P* with a maximal number of full components, such that each full component *C* is of one of the following four types. Let $m_C$ be the number of terminals in *C*. Then *C* consists of

- four edges, connected in a cross,
- a single complete segment with $m_C-2$ alternating incident edges,
- a corner and its legs, with $m_C-2$ alternating edges incident to a single leg, or
- a corner and its legs, with $m_C-3$ alternating edges incident to a single leg and a single edge incident to the other leg.

For all legs, the incident edge closest to the corner must point away from the opposite leg. Furthermore, the edges incident to the long leg on the same side as the short leg are at least as long as the short leg.

**Fig. 3:**
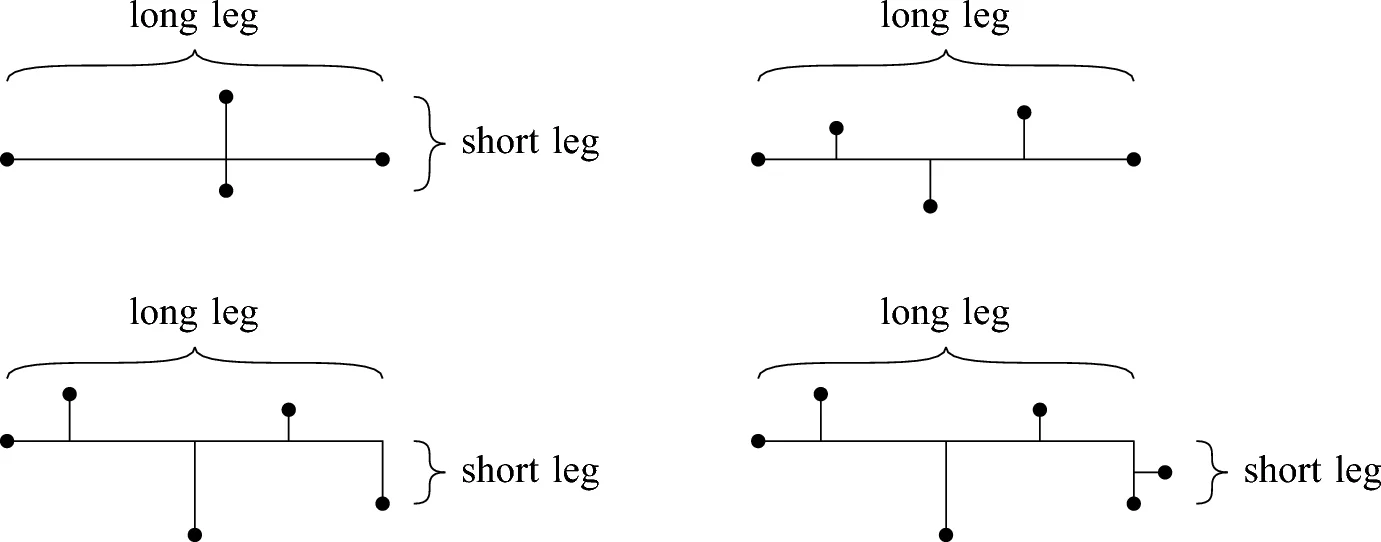
An example of each of the four different types of full components in Hwang trees

We will call MRSTs which have this property *Hwang trees*. See Figure 3 for an example of each of the four types of full components of Hwang trees.

**Fig. 4:**
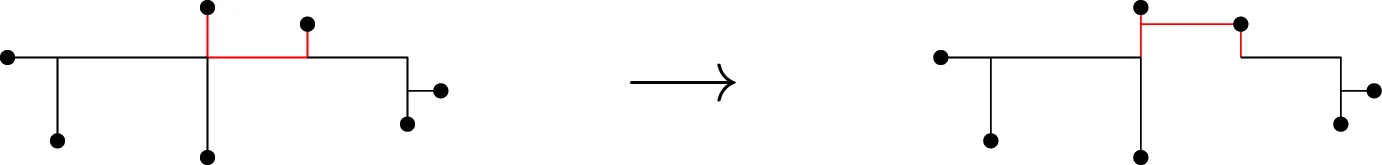
An example showing that Hwang trees do not contain a U-shape. The tree on the left has the same length as the tree on the right, but contains fewer full components. Therefore, the tree on the left is not a Hwang tree

Note that these full components do not contain a U-shape formed by an edge and two adjacent segments lying to the same side of that edge; any component with such a U-shape can be split into two full components by sliding the edge towards the terminals at the end of those segments. See Figure 4 for an example.

We will call the complete segment with the $m_C-2$ or $m_C-3$ incident edges the *long leg*, and the other leg (if any) the *short leg*. If there are two complete segments which both have $m_C-2$ or $m_C-3$ incident edges, we will consider the horizontal one to be the long leg, and the vertical one to be the short leg. If the long leg is horizontal (vertical), we call the full component a *horizontal (vertical) full component*.

## Minimum monotonic rectilinear Steiner trees

In this section, we present a simple algorithm which finds a minimum *monotonic* rectilinear Steiner tree (MMRST) in *O*(*n*) time. Then, we show under which circumstances an MMRST is an MRST. We also show that, as one might intuitively expect, an MMRST provides a good approximation of the MRST only if the strip is extremely narrow and sparse.

Let us start by finding an MMRST.

### Theorem 4

Let *P* be a set of *n* terminals in $\mathbb {R}^2$, sorted on *x*-coordinate. Then a minimum monotonic rectilinear Steiner tree can be found in *O*(*n*) time.

### Proof

We note that any two points that share an *x*-coordinate must be connected by a vertical line segment; otherwise, the separator directly to their left or to their right must be crossed at least twice. Therefore, the problem can be reformulated as the problem of finding an MMRST that contains a set $\{S_1,\ldots ,S_m\}$ of vertical line segments (possibly of zero length) with distinct *x*-coordinates, which is given ordered from left to right. We will now give a greedy algorithm to solve this problem.

The greedy algorithm goes over the segments $S_1,\ldots ,S_m$ in order. After handling a segment $S_i$, it has created a monotonic rectilinear spanning tree $T_i$ on the set $\{S_1,\ldots ,S_i\}$, which we will later prove to be of minimum length. Thus, after handling $S_m$ we have created an MMRST $T_m$ for the whole set $\{S_1,\ldots ,S_m\}$. The algorithm proceeds as follows. First, we set $T_1:= S_1$. Now assume we have handled $S_1,\ldots ,S_i$, and consider $S_{i+1}$. Let $\ell _i$ denote the vertical line containing $S_i$, and let $e_i:= T_i\cap \ell _i$. Note that if $S_i$ degenerates to a point, then $e_i$ could be a single point as well.

**Fig. 5:**
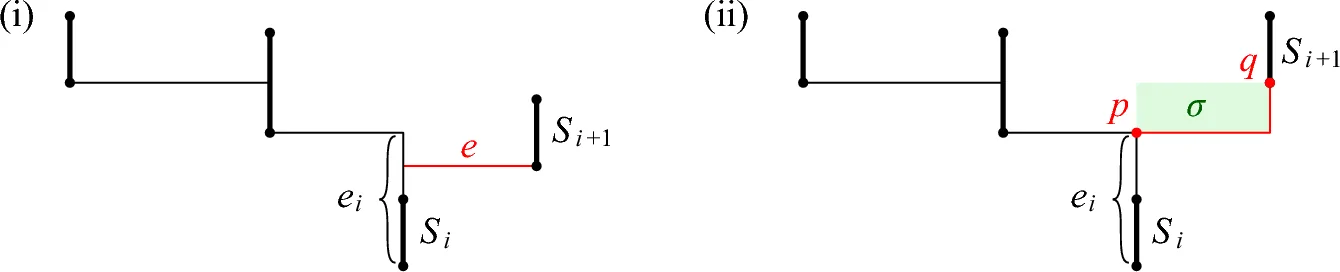
The two cases in the algorithm described in the proof of Theorem 4

- *Case (i): we can connect *$S_{i+1}$ *to *$e_i$ *with a single horizontal edge.* In this case we take an arbitrary such edge *e* and we set $T_{i+1}:= T_i \cup e \cup S_{i+1}$; see Figure 5(i).
- *Case (ii): we cannot connect *$S_{i+1}$ *to *$e_i$ *with a single horizontal edge.* Assume wlog that the lower endpoint *q* of $S_{i+1}$ lies above the horizontal line through the upper endpoint *p* of $e_i$. Then, $T_{i+1}$ is obtained by connecting $T_i$ to $S_{i+1}$ via the L-shape with vertices *p*, $(q_x,p_y)$, and *q*; see Figure 5(ii).

It is obvious that the algorithm runs in linear time, and that it constructs a spanning tree that is rectilinear and monotonic. To prove the correctness of the algorithm, we will show by induction on *i* that $T_i$ has the following property.

(∗): Let $T'_i$ be any (not necessarily minimum) monotonic rectilinear spanning tree on $\{S_1,\ldots ,S_i\}$, and let $e'_i:= T'_i\cap \ell _i$. Then $\Vert T'_i\Vert \geqslant \Vert T_i\Vert + \Vert e'_i \setminus e_i\Vert$.

Property (∗) essentially states that if the rightmost segment $e'_i$ of $T'_i$ is longer than the rightmost segment $e_i$ to $T_i$—this can make it cheaper to connect to $S_{i+1}$—then $T'_i$ has to pay for this with an increased total weight $\Vert T'_i\Vert$ (as compared to the total weight $\Vert T_i\Vert$ of $T_i$). Note that Property (∗) implies that $T_i$ is of minimum length, as the term $\Vert e'_i \setminus e_i\Vert$ is non-negative. Hence, proving Property (∗) will finish the proof.

Clearly, Property (∗) holds for $$T_1$$. Now assume Property (∗) holds for $$T_i$$ and consider $$T_{i+1}$$. Any monotonic rectilinear spanning tree $$T'_{i+1}$$ on $$\{S_1,\ldots ,S_{i+1}\}$$ consists of a tree $$T'_i$$ on $$\{S_1,\ldots ,S_{i}\}$$, a segment $$e'_{i+1}\supseteq S_{i+1}$$, and a path that connects $$T'_i$$ to $$e'_{i+1}$$. It will be convenient to assume that this path is a single horizontal segment $e^{\prime }$; this is wlog, since we can push the vertical edges of the path onto $$\ell _{i+1}$$. (Pushing the vertical segments onto $$\ell _{i+1}$$ can increase $$\Vert e'_{i+1} \setminus e_{i+1}\Vert $$, but this would only mean that we prove a stronger statement.) By the induction hypothesis, we have $$\Vert T'_i\Vert \geqslant \Vert T_i\Vert + \Vert e'_i \setminus e_i\Vert \geqslant \Vert T_i\Vert $$. In Case (i), we have $$\Vert T_{i+1}\Vert = \Vert T_i\Vert + \Vert e\Vert + \Vert S_{i+1}\Vert $$ and $$S_{i+1} = e_{i+1}$$, and so

$$ \begin{array}{lll} \Vert T'_{i+1}\Vert &  = &  \Vert T'_i\Vert + \Vert e' \Vert + \Vert e'_{i+1} \Vert \\ &  \geqslant &  \Vert T_i\Vert + \Vert e \Vert + \Vert S_{i+1} \Vert + \Vert e'_{i+1} \setminus S_{i+1} \Vert \\ &  = &  \Vert T_{i+1}\Vert + \Vert e'_{i+1} \setminus S_{i+1} \Vert \\ &  = &  \Vert T_{i+1}\Vert + \Vert e'_{i+1} \setminus e_{i+1} \Vert . \end{array} $$

For Case (ii), consider the rectangle $\sigma := [p_x,q_x]\times [p_y,q_y]$ as shown in Figure 5(ii). Since we assumed that $T'_{i+1}$ does not have vertical edges in the interior of σ, we have

$$ \Vert e'_i \cap \sigma \Vert + \Vert e'_{i+1} \cap \sigma \Vert = q_y-p_y = \Vert e_{i+1} \cap \sigma \Vert . $$

We thus have

$$ \begin{array}{lll} \Vert T'_{i+1}\Vert &  = &  \Vert T'_i\Vert + \Vert e' \Vert + \Vert e'_{i+1} \Vert \\ &  \geqslant &  \Vert T_i\Vert + \Vert e'_i \setminus e_i\Vert + \Vert e \Vert + \Vert e'_{i+1} \cap \sigma \Vert + \Vert e'_{i+1}\setminus \sigma \Vert \\ &  \geqslant &  \Vert T_i\Vert + \Vert e'_i \cap \sigma \Vert + \Vert e \Vert + \Vert e'_{i+1} \cap \sigma \Vert + \Vert e'_{i+1}\setminus \sigma \Vert \\ &  = &  \Vert T_i\Vert + \Vert e \Vert + \Vert e_{i+1} \cap \sigma \Vert + \Vert e'_{i+1} \setminus \sigma \Vert \\ &  = &  \Vert T_i\Vert + \Vert e \Vert + \Vert e_{i+1} \Vert - \Vert S_i\Vert + \Vert e'_{i+1} \setminus \sigma \Vert \\ &  = &  \Vert T_{i+1}\Vert + \Vert e'_{i+1} \setminus e_{i+1} \Vert . \end{array} $$

This finishes the proof that Property (∗) holds and, hence, the proof of the lemma. □

Now we have found an MMRST, we also show when it is optimal.

### Theorem 5

Let *P* be a point set in a δ-strip. If $(x_i = x_j$ or $x_j - x_i \geqslant \delta )$ for all i<j, then an MMRST on *P* is an MRST on *P*. The threshold δ on the difference between the *x*-coordinates is tight in the worst case.

### Proof

First, we show that if for all i<j we have $$x_i = x_j$$ or $$x_j - x_i \geqslant \delta $$, we can make any MRST monotonic without making it longer. Let *T* be an MRST on *P*. Do the following exhaustively. Let $$e_1$$ and $$e_2$$ be any two horizontal edges of *T* showing that *T* is not monotonic, i.e., the horizontal intervals covered by the edges (partially) overlap in more than a single point. Then we apply the same procedure as used in the proof of Observation 2 to $$e_1$$ and $$e_2$$, where we take ℓ and $\ell^{\prime }$ to be the leftmost and rightmost vertical line, respectively, that intersect $$e_1$$ as well as $$e_2$$. Note that ℓ and $\ell^{\prime }$ are lines of the Hanan grid, and that dist$$(\ell ,\ell ') \geqslant \delta $$ by our assumption. Let us call the resulting tree $T^{\prime }$. Note that the total length of $T^{\prime }$ is $$\Vert T\Vert - (x_j-x_i)+ |y'_2-y'_1 |\leqslant \Vert T\Vert -\delta +\delta = \Vert T\Vert $$. Therefore, $T^{\prime }$ is an MRST. Finally, we note that we can indeed do the above exhaustively, since $T^{\prime }$, when seen as a subset of the Hanan grid, uses strictly fewer horizontal edges than *T*. We conclude that if for all i<j we have $$x_i = x_j$$ or $$x_j - x_i \geqslant \delta $$, then an MMRST is indeed an MRST.

**Fig. 6:**
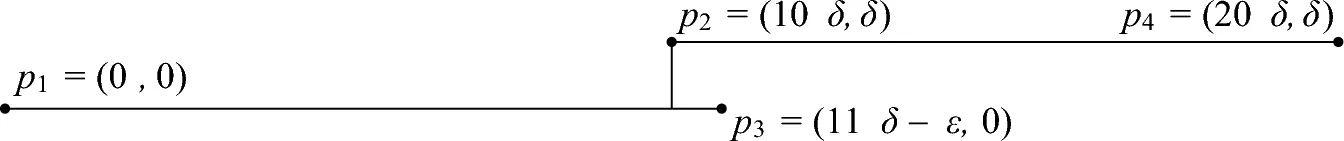
A point set for which no MRST is monotonic. (In black, a non-monotonic MRST)

Next, we show that the threshold δ is tight, that is, we show that for any ε>0 there is a point set such that $x_j - x_i \geqslant \delta -\varepsilon$ for all i<j, while there is no monotonic MRST. Consider the point set *P* shown in Figure 6, consisting of the following four points: $p_1 = (0, 0), p_2 = (10\,\delta , \delta ), p_3 = (11\,\delta -\varepsilon ,0)$ and $p_4 = (20\,\delta ,\delta )$. Now, an MMRST has total length 22δ, but an MRST has total length $22\delta -\varepsilon$. □

We finish by giving an upper bound to the approximation factor of an MMRST. Recall that a point set *P* is called *k-sparse* if for all *x*, the rectangle $[x,x+1] \times [0,\delta ]$ contains at most *k* points for some sparseness parameter k⩾1.

### Theorem 6

Let δ>0. Let *P* be a *k*-sparse point set, let $\text{ opt }(P)$ denote the length of an MRST on *P*, and let $\text{ opt}_{\textrm{mon}}(P)$ denote the length of an MMRST on *P*. Then $\text{ opt}_{\textrm{mon}}(P)= O(k\delta )\cdot \text{ opt }(P)$. Moreover, there are *k*-sparse point sets *P* such that $\text{ opt}_{\textrm{mon}}(P)= \Omega (k\delta )\cdot \text{ opt }(P)$.

### Proof

Let $\delta ' :=\max _i y_i - \min _i y_i$ be the maximal height difference between any two points. A simple lower bound on the length of an MRST on *P* is $\delta ' + x_n - x_1$, and a simple upper bound is $(n-1)\delta ' + x_n - x_1$. Therefore, an MMRST is an α-approximation of an MRST for

$$ \alpha = \frac{(n-1)\delta ' + x_n - x_1}{\delta ' + x_n - x_1} < \frac{(n-1)\delta + \lfloor (n-1)/k \rfloor }{\delta + \lfloor (n-1)/k \rfloor } = O(\delta k). $$

**Fig. 7:**
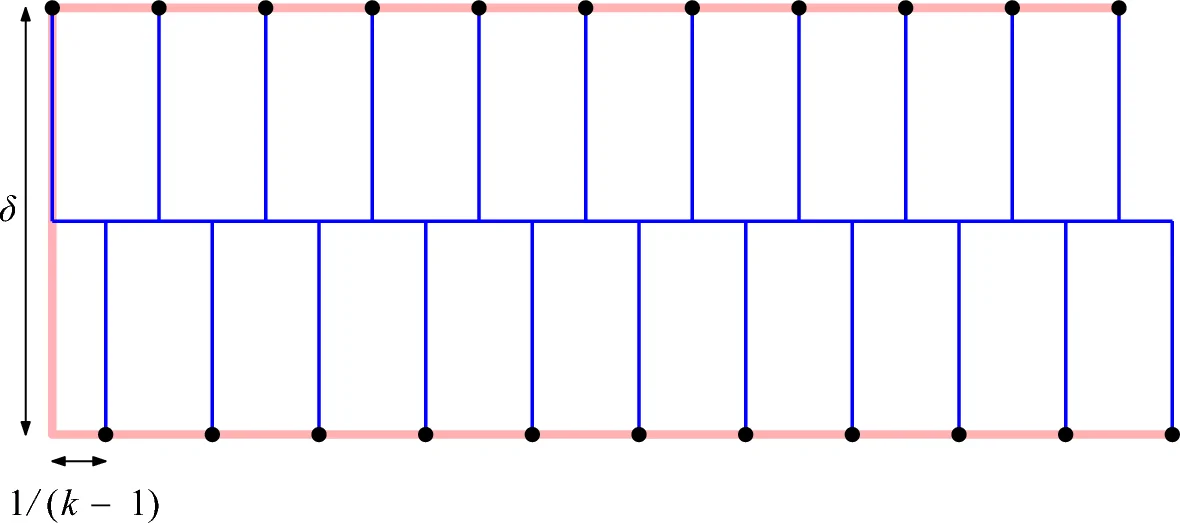
Example used in the proof of Theorem 6. An MMRST is shown in blue, and a non-monotonic rectilinear Steiner tree is shown in pink

It remains to observe that an approximation factor of $\Omega (\delta k)$ can indeed be reached. To see this, let *P* be a point set with $x_i=i/(k-1)$, and $y_i = \delta$ if *i* is even and $y_i=0$ if *i* is odd, where we assume for simplicity that k⩾2; see Figure 7.

Then the length of an MMRST is $(n-1)\cdot \tfrac{1}{k-1} + n\cdot \tfrac{\delta }{2}$, while an MRST has a length of less than $\delta + \tfrac{2(n-1)}{k-1}$. For $n \rightarrow \infty$, this results in an approximation ratio of at least $\tfrac{1}{2} + \tfrac{\delta (k-1) }{4} =\Omega (k\delta )$. □

## Sparse point sets inside a narrow strip

We say that a point set is *k-sparse* if for all *x* the rectangle $[x,x+1] \times [0,\delta ]$ contains at most *k* points for some *sparseness parameter* *k*. In this section, we will give an $n^{O(k\sqrt{\delta })}$ algorithm to compute an MRST for *k*-sparse point sets, using separators. We will do so in two steps. First, we will show that all separators are crossed at most $O(k\sqrt{\delta })$ times. Then, we will give a dynamic-programming algorithm which sweeps from left to right and runs in the desired time. After doing so, we will prove two further properties of the given algorithm. First, we show how to turn the algorithm into a (1+ε)-approximation algorithm that runs in $(k/\varepsilon )^{O(k\sqrt{\delta }\log \delta )}n$ time. Second, we will show that any strictly faster algorithm will need to apply a significantly different approach.

We start by bounding the tonicity at the separators. The following lemma will also be useful later, when we deal with randomly generated point sets.

### Lemma 7

Let *T* be a Hwang tree on *P*. Let $s_i$ be a separator such that

2 $$\begin{aligned} x_{i+\lceil \sqrt{\delta } \rceil +c_1} - x_{i} > c_2 \sqrt{\delta }\end{aligned}$$

for an integer constant $c_1 \geqslant 0$ and a constant $c_2 > 0$. Then the tonicity of *T* at $s_i$ is $O(\sqrt{\delta }+1)$.

### Proof

We will show that *T* crosses $s_i$ at most $17 c_1 + 36 + (4/c_2 + 17) \sqrt{\delta }$ times. Recall that $P[i,j] :=\{p_i,\ldots ,p_j\}$ and define $P' :=P[i,i+\lceil \sqrt{\delta } \rceil +c_1]$. The edges of *T* crossing $s_i$ can be split into five sets:

- $E_{\textrm{long}}$, the set of edges which also cross the vertical line defined by $x = x_i + c_2 \sqrt{\delta }/ 2$.
- $E_{\textrm{h}}$, the set of edges not in $E_{\textrm{long}}$ that are part of a horizontal full component.
- $E_{\textrm{vl}}$, the set of edges not in $E_{\textrm{long}}$ that are part of a vertical full component whose long leg lies to the left of $s_i$.
- $E_{\textrm{vb}}$, the set of edges not in $E_{\textrm{long}}$ that are part of a vertical full component whose long leg lies between $s_i$ and the vertical line $x = x_i + c_2 \sqrt{\delta }/2$.
- $E_{\textrm{vr}}$, the set of edges not in $E_{\textrm{long}}$ that are part of the short leg of a vertical full component whose long leg lies to the right of the vertical line $x = x_i + c_2 \sqrt{\delta }/ 2$.

**Fig. 8:**
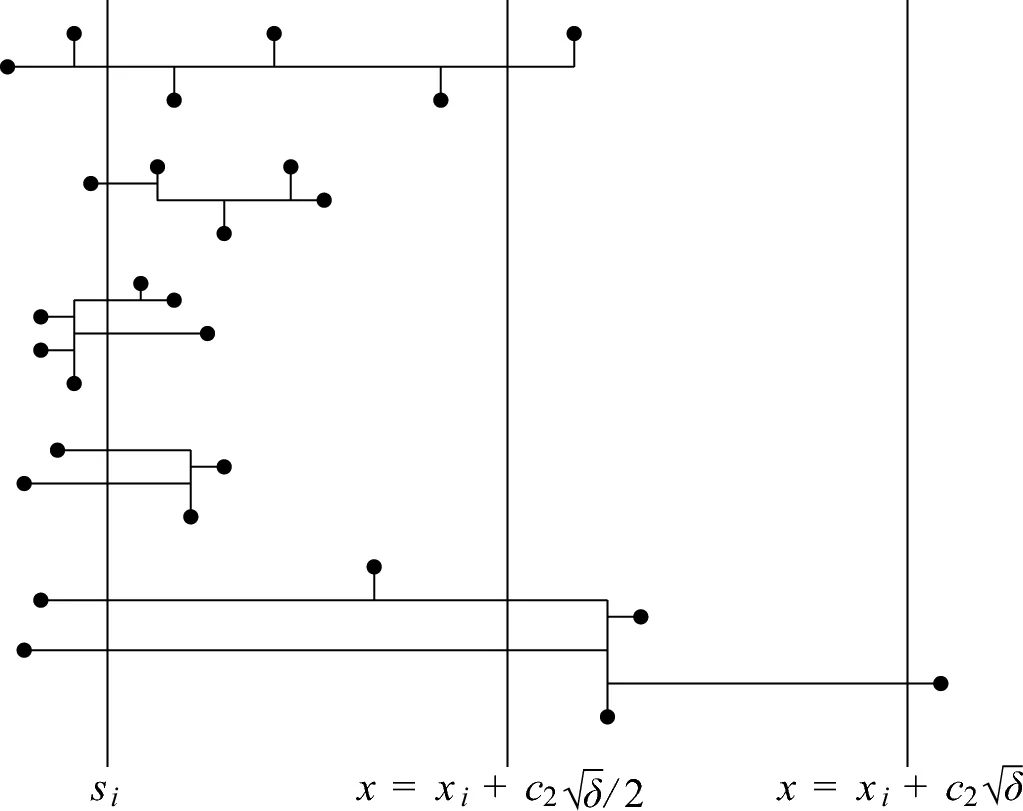
Examples of the different types of edges crossing $s_i$, not to scale. Topmost, two edges in $E_{\textrm{h}}$, one as part of the long leg, and one as the incident edge of the short leg of a horizontal full component. Then, two edges in $E_{\textrm{vl}}$, one as part of the short leg, and one as an edge incident to the long leg. Third, two edges in $E_{\textrm{vb}}$, one as the short leg, and one as an edge incident to the long leg. Finally, an edge in $E_{\textrm{vr}}$ and an edge in $E_{\textrm{long}}$

See Figure 8 for examples.

By Observation 2, we have

3 $$\begin{aligned} |E_{\textrm{long}} |\leqslant 1+\left\lfloor \delta / \left( c_2 \sqrt{\delta }/ 2 \right) \right\rfloor \leqslant 1+2 \sqrt{\delta }/ c_2. \end{aligned}$$

Secondly, every horizontal full component corresponding to a edge in $$E_{\textrm{h}}$$ contains a terminal from $P^{\prime }$. Since horizontal full components cross $$s_i$$ at most once and all terminals are part of at most four full components, we conclude that

4 $$\begin{aligned} |E_{\textrm{h}}|\leqslant 4 |P'|. \end{aligned}$$

The right endpoints of edges in $$E_{\textrm{vl}}$$ are either terminals in $P^{\prime }$ or T-points incident to a vertical edge whose other endpoint is a terminal in $P^{\prime }$. Since all terminals are part of at most four full components, we conclude that

5 $$\begin{aligned} |E_{\textrm{vl}}|\leqslant 4 |P'|. \end{aligned}$$

To bound $|E_{\textrm{vb}}|$, let $C_{\textrm{vb}}$ be the set of all full components with at least one edge in $E_{\textrm{vb}}$. For a component $C \in C_{\textrm{vb}}$, let *S*(*C*) be the set of horizontal complete segments to the right of the long leg of *C*. Recall that the segments incident to the long leg of a vertical full component *C* alternate between lying to the right and left of the long leg. Hence, the number of edges in $E_{\textrm{vb}}$ from *C* is bounded by |S(C)|+1, and so

6 $$\begin{aligned} |E_{\textrm{vb}}|\leqslant |C_{\textrm{vb}}|+ \sum _{C \in C_{\textrm{vb}}} |S(C)|. \end{aligned}$$

Note that for any component $$C \in C_{\textrm{vb}}$$, the terminal incident to its long leg must be a point in $P^{\prime }$. Furthermore, every point in $P^{\prime }$ can only be used twice this way. Therefore, $$|C_{\textrm{vb}}|\leqslant 2 |P'|$$.

To bound $$\sum _{C \in C_{\textrm{vb}}} |S(C)|$$, we note that each segment in any set *S*(*C*) either (i) is a short leg, (ii) ends in a point of $P^{\prime }$, or (iii) crosses the vertical line $$x = x_i + c_2 \sqrt{\delta }$$. Since $$|C_{\textrm{vb}}|\leqslant 2 |P'|$$, there are at most $2|P^{\prime }|$ segments of type (i). Trivially, there are at most $|P^{\prime }|$ segments of type (ii). Finally, by Observation 2, we can only have $$1+\lfloor \delta / (c_2 \sqrt{\delta }/ 2) \rfloor \leqslant 1+2 \sqrt{\delta }/ c_2$$ segments of type (iii). Therefore,

7 $$\begin{aligned} \sum _{C \in C_{\textrm{vb}}} |S(C)|\leqslant 3 |P'|+ 1+2 \sqrt{\delta }/ c_2.\end{aligned}$$

We conclude that

8 $$\begin{aligned} |E_{\textrm{vb}}|\leqslant 2|P'|+ 3 |P'|+ 1+2 \sqrt{\delta }/ c_2 = 5|P'|+ 1+2 \sqrt{\delta }/ c_2.\end{aligned}$$

Finally, the right endpoints of edges in $$E_{\textrm{vr}}$$ are T-points incident to a vertical edge whose other endpoint is a terminal in $P^{\prime }$. Since all terminals are part of at most four full components,

9 $$\begin{aligned} |E_{\textrm{vr}}|\leqslant 4 |P'|.\end{aligned}$$

Since $|P'|=\big \lceil \sqrt{\delta } \big \rceil +c_1+1$, the total number of edges crossing $s_i$ can now be bounded by

$$\begin{aligned} |E_{\textrm{long}} |+ |E_{\textrm{h}} |+ |E_{\textrm{vl}} |+ |E_{\textrm{vb}} |+ |E_{\textrm{vr}} |&\leqslant 17 |P'|+ 2(1+2 \sqrt{\delta }/ c_2) \\&= 17 \left( \big \lceil \sqrt{\delta } \big \rceil +c_1+1 \right) + 2+4 \sqrt{\delta }/ c_2 \\&\leqslant 17 c_1 + 36 + (4/c_2 + 17) \sqrt{\delta }. \end{aligned}$$

□

Using Lemma 7 we can now prove a bound on the tonicity of MRSTs of sparse point sets.

### Corollary 8

An MRST on a *k*-sparse point set *P* in a δ-strip is $\bigl ((9k+18)(2+ \sqrt{\delta })\bigr )$-tonic.

### Proof

First, we note that since our point set *P* is *k*-sparse, we have $x_j - x_i \geqslant \lfloor (j-i)/k \rfloor$ for all j>i. Specifically, for all $s_i$ such that $i+\lceil \sqrt{\delta } \rceil +k \leqslant n$, we get

10 $$\begin{aligned} x_{i+\big \lceil \sqrt{\delta } \big \rceil +k} - x_i \geqslant \left\lfloor \frac{\big \lceil \sqrt{\delta } \big \rceil +k}{k} \right\rfloor \geqslant \left\lfloor \frac{\sqrt{\delta }}{k}+1 \right\rfloor \geqslant \frac{\sqrt{\delta }}{k}.\end{aligned}$$

Therefore, we can invoke Lemma 7 with $c_1 = k$ and $c_2 = 1/k$, giving us that all these $s_i$ are crossed at most $17k + 36 + (4k + 17) \sqrt{\delta }$ times. (These constant follow from the constants in the proof of Lemma 7, see above.) By symmetry, we can do the same for all $s_i$ such that $(i+1)-\lceil \sqrt{\delta } \rceil -k \geqslant 1$. Finally, we note that if this does not cover all $s_i$, then we have fewer than $17k + 36 + (4k + 17) \sqrt{\delta }$ points in total. Since every separator is crossed at most *n* times, the statement also holds in this case. We conclude that for sparse terminal sets, all separators are crossed at most $17k + 36 + (4k + 17) \sqrt{\delta }< (9k+18)(2+ \sqrt{\delta })$ times. □

**Fig. 9:**
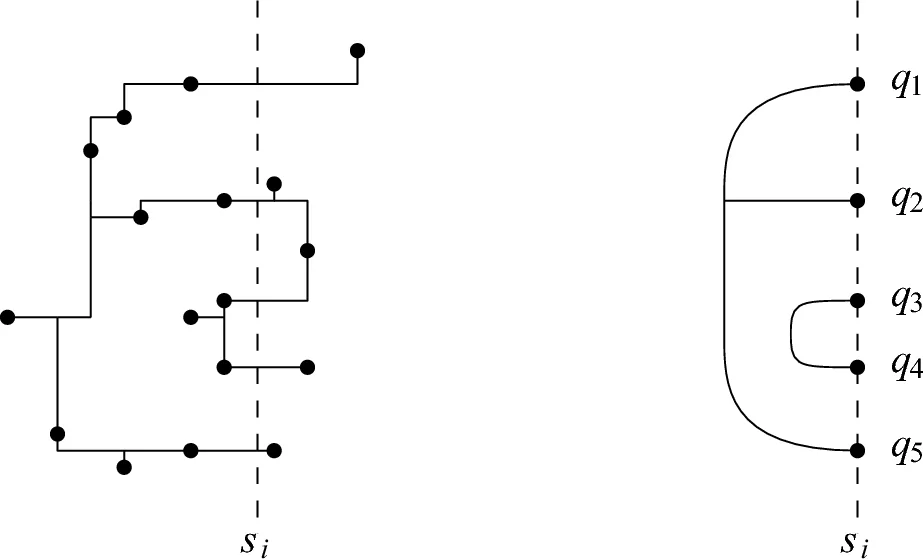
An example of a rectilinear Steiner tree and its crossing pattern $C = \{ \{q_1,q_2,q_5\}, \{q_3,q_4\} \}$ at $s_i$

Corollary 8 gives rise to a natural dynamic-programming algorithm, as explained next. Let *T* be a rectilinear Steiner tree, and let $s_i$ be a separator. We define the crossingpattern of *T* at $s_i$ as follows. Let $X(s_i)$ be the set of at most *n* points where the Hanan grid crosses $s_i$, and let $X(s_i,T) \subseteq X(s_i)$ be the subset of points where *T* crosses $s_i$. If *T* is an MRST,

11 $$\begin{aligned} |X(s_i,T)|\leqslant (9k+18)(2+ \sqrt{\delta })= O(k\sqrt{\delta }) \end{aligned}$$

by Corollary 8. We partition $X(s_i,T)$ into parts (that is, subsets) such that two points from $X(s_i,T)$ are in the same part if the path in *T* between these points fully lies to the left of $s_i$. The resulting partition of $X(s_i,T)$ is the crossing pattern of *T* at $s_i$; see Figure 9 for an example.

We will say that a rectilinear forest *T* *adheres to* a crossing pattern *C* at $$s_i$$ if *T* lies fully to the left of $$s_i$$, and there exists a rectilinear forest $T^{\prime }$ which lies fully to the right of $$s_i$$ such that $T\cup T^{\prime }$ is a rectilinear Steiner tree with crossing pattern *C* at $$s_i$$. Note that not all crossing patterns can lead to an MRST: those that require crossing edges on the left-hand side (because they do not have a proper ‘nesting structure’) can never lead to an MRST. We call the crossing patterns that contain at most $$(9k+18)(2+ \sqrt{\delta })$$ points and do not require crossing edges on the left-hand side *viable crossing patterns*. We will now count the number of viable crossing patterns at $$s_i$$. There are $$n^{O(k\sqrt{\delta })}$$ possible sets $$X(s_i,T)$$ that contain at most $$(9k+18)(2+ \sqrt{\delta })$$ points. The number of viable partitions of these points—also known as the number of non-crossing partitions—follows the Catalan numbers. Hence, there are $$2^{O(k\sqrt{\delta })}$$ possible viable partitions for each $$X(s_i,T)$$. This implies that the total number of viable crossing patterns for $$s_i$$ is $$n^{O(k\sqrt{\delta })} \cdot 2^{O(k\sqrt{\delta })} = n^{O(k\sqrt{\delta })}$$.

**The algorithm.** We can now define a table entry *A*[*i*, *X*] for each separator $s_i$ and viable crossing pattern *X* at $s_i$ as follows.

*A*[*i*, *X*] := the minimum length of a rectilinear forest adhering to *X* at $s_i$.

Note that the length of an MRST equals $A[n,\{\emptyset \}]$. Next we describe a recursive formula to compute the table entries. As a base case, we will use A[0,X]=0 for $X = \{\emptyset \}$, and ∞ for all other *X*. For simplicity, we will for now assume that all *x*-coordinates are distinct. Later, we will argue that it is easy to adapt the argument to the general case.

Let $$F(X,s_i)$$ be a minimum-length rectilinear forest adhering to *X* at $$s_i$$, and let $X^{\prime }$ be its (unknown) crossing pattern at $$s_{i-1}$$. Then the value of *A*[*i*, *X*] equals the value of $A[i-1,X^{\prime }]$ plus the total length of the edges of $$F(X,s_i)$$ between $$s_{i-1}$$ and $$s_i$$. The total length of $$F(X,s_i)$$ between these two separators only depends on $X^{\prime }$ and *X*. Since this subproblem contains $$O(k\sqrt{\delta })$$ points with three different *x*-coordinates, its Hanan grid contains only $$O(k\sqrt{\delta })$$ edges. Therefore, its value can be computed in $$2^{O(k\sqrt{\delta })}$$ time by simply checking every possible subset of edges. Let $L(X^{\prime },X)$ denote the total length of the solution to this subproblem. If no solution exists, we define it to be ∞. Then we get

12 $$\begin{aligned} A[i,X] = \min _{\text {viable }X'} A[i-1,X'] + L(X',X). \end{aligned}$$

So far, we assumed that all *x*-coordinates are distinct. In the general case, we skip over all non-existent $s_i$. So, let $s_j$ and $s_i$ be consecutive separators, with j<i. Note that since the point set is sparse, at most *k* points share an *x*-coordinate. Therefore, j⩾i-k. We get

13 $$\begin{aligned} A[i,X] = \min _{\text {viable }X_j} A[j,X_j] + L(X_j,X_i), \end{aligned}$$

where $L(X_j,X_i)$ is defined analogously to $L(X_{i-1},X_i)$. Note that the Hanan grid of the corresponding subproblem still has at most $O(k\sqrt{\delta })$ edges. Therefore, $L(X_j,X_i)$ can still be computed in $2^{O(k\sqrt{\delta })}$ time.

**The running time.** To analyse the running time, we first determine the number of table entries. There are *O*(*n*) separators, and we have already seen for every separator $$s_i$$ there are $$n^{O(k\sqrt{\delta })}$$ possible viable crossing patterns. Hence, the total number of table entries is $$O(n) \cdot n^{O(k\sqrt{\delta })} = n^{O(k\sqrt{\delta })}$$. Next, we calculate the time needed per table entry. For each of the $$n^{O(k\sqrt{\delta })}$$ possible viable crossing patterns $X^{\prime }$ we compute $L(X^{\prime },X)$ in $$2^{O(k\sqrt{\delta })}$$ time. This brings the total time needed per table entry to $$n^{O(k\sqrt{\delta })}$$. Since we have $$n^{O(k\sqrt{\delta })}$$ table entries, each needing $$n^{O(k\sqrt{\delta })}$$ time, we obtain the following theorem.

### Theorem 9

Let *P* be a *k*-sparse point set of size *n* inside a δ-strip. Then we can compute an MRST on *P* in $n^{O(k\sqrt{\delta })}$ time.

We continue this section by showing that this algorithm has two further properties. First, we show how to turn this algorithm into an approximation algorithm. Second, we show that there can indeed be many different viable crossing patterns. Therefore, any strictly faster algorithm will need a significantly different approach.

### Theorem 10

Let *P* be a *k*-sparse point set of size *n* inside a δ-strip. Assuming the points in *P* are already sorted by *x*-coordinate, we can compute an (1+ε)-approximation of the MRST on *P* in $(k/\varepsilon )^{O(k\sqrt{\delta }\log \delta )} n$ time.

### Proof

The algorithm consists of four simple steps. If |P|⩽2k then we compute an MRST in $$k^{O(\sqrt{k})}$$ time [11] time. Otherwise, for every point in *P*, we round its *y*-coordinate to the nearest multiple of $$\zeta :=\varepsilon / (2k)$$, creating a set $P^{\prime }$. Then, we run our MRST algorithm on $P^{\prime }$. Finally, for every point $p^{\prime }$ in $P^{\prime }$, we connect $p^{\prime }$ and the corresponding point in *P* if necessary.

First, we show that this indeed results in a (1+ε)-approximation. Clearly, if |P|⩽2k, this is indeed the case. Otherwise, we start by noting that the MRST of $P^{\prime }$ is at most nζ/2 longer than the MRST of *P*. After all, we can create a (not necessarily minimum) rectilinear Steiner tree of $P^{\prime }$ by taking the MRST of *P*, and then adding edges between every point $p^{\prime }$ of *P* and its corresponding point in *P* where necessary. Since we add at most a total of nζ/2 length of edges to the MRST of $P^{\prime }$, the solution returned is at most nζ longer than the MRST of *P*. Next, we note that the minimum length of the MRST of *P* is $$x_n - x_1 \geqslant \lfloor (n-1)/k \rfloor \geqslant n/(2k)$$. Therefore, the rectilinear Steiner tree we return is an α-approximation for $$\alpha =\frac{n/(2k) + n \zeta }{n/(2k)} = (1 + 2k \zeta ) = (1+\varepsilon )$$.

The proof of the running time is analogous to the proof of the running time of the algorithm used, with one exception: the dependency on *n*. Instead of *O*(*n*) possible *y*-coordinates, we have only $O(\delta /\zeta )$. Therefore, there are only $(\delta /\zeta )^{O(k\sqrt{\delta })} = (1/\zeta )^{O(k\sqrt{\delta }\log \delta )}$ possible viable crossing patterns. Combining this with the facts that we sweep from left to right and that we assume the points are already sorted, we conclude that the dependency on *n* is linear. All in all, the total running time is

$$ (1/\zeta )^{O(k\sqrt{\delta }\log \delta )} n = (k/\varepsilon )^{O(k\sqrt{\delta }\log \delta )}n. $$

□

Finally, we show that there are indeed many different viable crossing patterns, even for 2-sparse point sets.

### Proposition 11

Let *n* be large enough. Then there exist a function *f*, a 2-sparse point set *P* of *n* points and a set $\mathcal {P}$ of $n^{\Theta (\sqrt{\delta })} f(\delta )$ 2-sparse point sets $P_i$ of $\Theta (\sqrt{\delta })$ points that lie fully to the right of *P* such that the following holds:

- for each *i*, all MRSTs on $P \cup P_i$ have the same crossing pattern at $s_n$, and
- for each *i*, *j* with i≠j, the crossing pattern at $s_n$ of an MRST on $P\cup P_i$ is different from the crossing pattern at $s_n$ of an MRST on $P\cup P_j$ .

### Proof

**Fig. 10:**
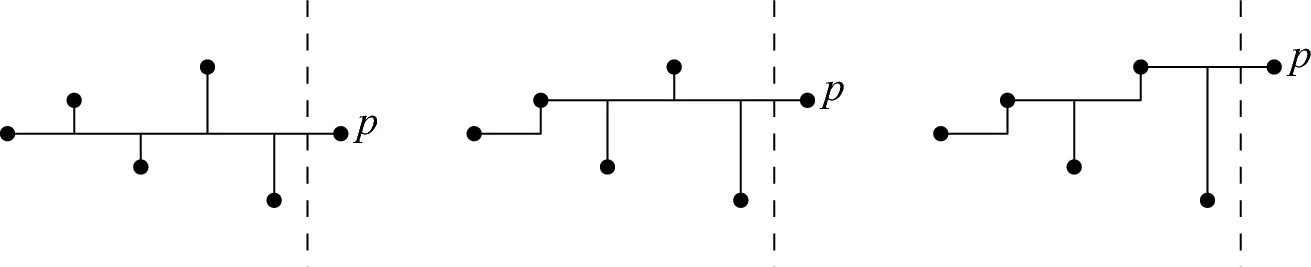
An example of a hook and three of the five resulting crossing patterns (*y*-coordinates exaggerated for visibility)

We start by introducing a gadget, which we will call a *hook*. A hook *H* is a set of *m* points in a ‘<’-form, where the points (ordered from left to right) alternately are above the highest point and below the lowest point so far. See Figure 10 for an example. Suppose we add a point *p* which is to the right of *H* and has a *y*-coordinate used by a point of *H*. Then, any MRST on the set $H \cup \{p\}$ contains an edge from *p* going left. Therefore, we can use a hook of *m* points to generate *m* different crossing patterns. Note that the difference in *y*-coordinates of the points can be arbitrarily small, so we will treat them as such.

**Fig. 11:**
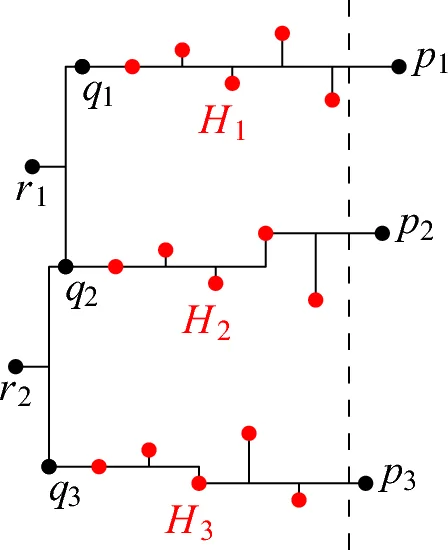
An example of a point set $P \cup P_i$. The set *P* lies to the left of the separator, the three hooks of *P* are shown in red, and $P_i = \{p_1,p_2,p_3\}$ lies to the right of the separator. The image is scaled for visibility; the vertical distance between two hooks is approximately 100 times larger than the horizontal distance between two points of a hook

We will now place $\Theta (\sqrt{\delta })$ hooks containing $\Theta (n / \sqrt{\delta })$ points each below each other. If these hooks act independently, we can choose $\Theta (n/\sqrt{\delta })$ different *p* for each of the $\Theta (\sqrt{\delta })$ hooks, resulting in the required $\Theta (n / \sqrt{\delta })^{\Theta (\sqrt{\delta })} = n^{\Theta (\sqrt{\delta })} f(\delta )$ crossing patterns. To do so, we connect the hooks on the left hand side. See Figure 11 for an example.

To be precise, suppose we have $m = \lceil \sqrt{\delta }/ 10 \rceil$ hooks $H_1, \ldots , H_m$ from top to bottom. To ensure the point set is 2-sparse, all points will have distinct integer *x*-coordinates. We distribute these equally such that for each hook, the horizontal distance between two consecutive points is *m*. The offsets are such that $H_i$ is more to the right than $H_j$ if i<j. Note that the vertical distance between two hooks is approximately $\delta / (m-1) \approx 10 \sqrt{\delta }\approx 100m$. To the left of each hook $H_i$ we add an extra point $q_i$ on the same height of the leftmost point of $H_i$. Finally, for each i<m, we add a point $r_i$ to the left of $H_i$ with a height halfway between those of the leftmost points of $H_i$ and $H_{i+1}$. These points together form the point set *P*.

Now, all that remains is to show that the hooks indeed act independently. This is easy to see; as the vertical distance between two hooks is a factor 100 larger than the horizontal distance between two consecutive points in the same ‘row’, the vertical distance between two consecutive hooks will be bridged exactly once. Since there must be a horizontal segment between the leftmost point of every $H_i$ and the corresponding $q_i$, every $r_i$ is connected to $q_i$ and $q_{i+1}$. Since the hooks are guaranteed to be connected on the left hand side, each of the $n^{\Theta (\sqrt{\delta })} f(\delta )$ different combinations of points $p_1, \ldots , p_m$ guarantees a different crossing pattern, as required. □

## Random point sets inside a narrow rectangle

In this section, we give an algorithm with $\min \{n^{O(\sqrt{\delta })}, 2^{O(\delta \sqrt{\delta })} n\}$ expected running time for random point sets. Specifically, we assume the points in *P* are taken independently uniformly at random from the rectangle $[0,n] \times [0, \delta ]$.

Recall that the algorithm for sparse point sets from the previous section, which had running time $n^{O(\sqrt{\delta })}$, was based on the fact that any separator $s_i$ of a sparse point set is crossed only $O(\sqrt{\delta })$ times. Thus for each separator there are $n^{O(\sqrt{\delta })}$ different crossing patterns. Our main goal is now to develop an algorithm for random point sets that is fixed-parameter tractable with parameter δ. To obtain such algorithm we face three difficulties. For the first problem, let us define the *spacing* of $p_i$ (in *P*) as $\Delta _i = x_{i+1} - x_i$, for all $1 \leqslant i \leqslant n-1$. We extend this definition with $\Delta _0 = x_1$, and $\Delta _n = n - x_n$. Note that $\sum _{i=0}^n \Delta _i = n$. The values $\Delta _i$ play an important role in the analysis of the algorithm, and it will be convenient to assume that the $\Delta _i$ are independent. However, when the *x*-coordinates of the points are generated uniformly at random in the interval [0, *n*], this is not quite true. Second, unlike in the case of sparse point sets, we cannot guarantee that all separators are crossed only $O(\sqrt{\delta })$ times. Third, even if a separator is crossed $O(\sqrt{\delta })$ times, the number of candidate crossing patterns can still be $n^{\Theta (\sqrt{\delta })}$, which is too much for an FPT algorithm. We overcome these difficulties as follows.

For our first problem, the dependent spacings $\Delta _i$, we will use a recent lemma [1, Lemma 5.5], as explained next. Let $X_{\delta ,n}$ be a random point set of *n* points, where the points are taken independently uniformly from $[0, n] \times [0,\delta ]$. Let $Y_{\delta ,n}$ be a random point set of *n* points, where the spacings $\Delta _i = x_{i+1}-x_i$ are taken independently from $\textrm{Exp}(1)$, and the *y*-coordinates are taken independently uniformly from [0,δ]. We then get:

### Lemma 12

(Alkema *et al.* [1]) Let $X_{\delta ,n}$ and $Y_{\delta ,n}$ be as defined above. Let $\mathcal {A}$ be an algorithm that runs on point sets in $\mathbb {R}^2$. Let $T_\mathcal {A}(P)$ denote its running time for a point set *P*. Suppose for all λ>1 and *P*, we have $T_\mathcal {A}(P_\lambda ) \leqslant T_\mathcal {A}(P)$, where $P_\lambda$ is obtained from *P* by scaling the *x*-coordinates by a factor λ>1. Suppose $\mathbb {E}[T_\mathcal {A}(Y_{\delta ,n})] = O(f(\delta ,n))$ for some function *f*. Then $\mathbb {E}[T_\mathcal {A}(X_{\delta ,n})] = O(f(\delta ,n))$.

This gives us the desired independence of the $\Delta _i$. Therefore, we will from now on assume the point set is generated following the distribution $Y_{\delta ,n}$.

To deal with the second issue we will define a certain configuration of points and a corresponding separator—we will call such separator a *soft wall*—such that the separator is crossed only $O(\sqrt{\delta })$ times. Our new dynamic programming algorithm will have table entries for every soft wall instead of for every separator. We will prove that we expect to find sufficiently many soft walls, so that the expected number of points in between two consecutive soft walls only depends on δ (and not on *n*).

This still leaves the third problem, because where a soft wall is crossed by an MRST may depend on points from *P* that are beyond the previous or next soft wall. Thus the number of crossing patterns can still be $n^{\Theta (\sqrt{\delta })}$. We therefore also devise a second type of wall, the *hard wall*. This is a vertical line ℓ through an input point $p_i$ that will not be crossed at all by any edge of an MRST. The MRST will consist of two independent parts: an MRST for the points to the left of ℓ plus $p_i$ itself, and an MRST for the points to the right of ℓ plus $p_i$ itself. More generally, if we have a collection of hard walls then the subproblems between any two consecutive hard walls are completely independent. Hard walls will occur much less frequently than soft walls, but still the expected number of points in between two consecutive hard walls will be shown to depend only on δ. Hence, the number of crossing patterns we need to consider for the soft walls in between the two hard walls only depends on δ, giving us an FPT algorithm.

Before we describe these steps in more detail, we first give pseudocode for the global algorithm. Recall that $P[i,j] :=\{p_i,\ldots ,p_j\}$.

**Algorithm 1:**
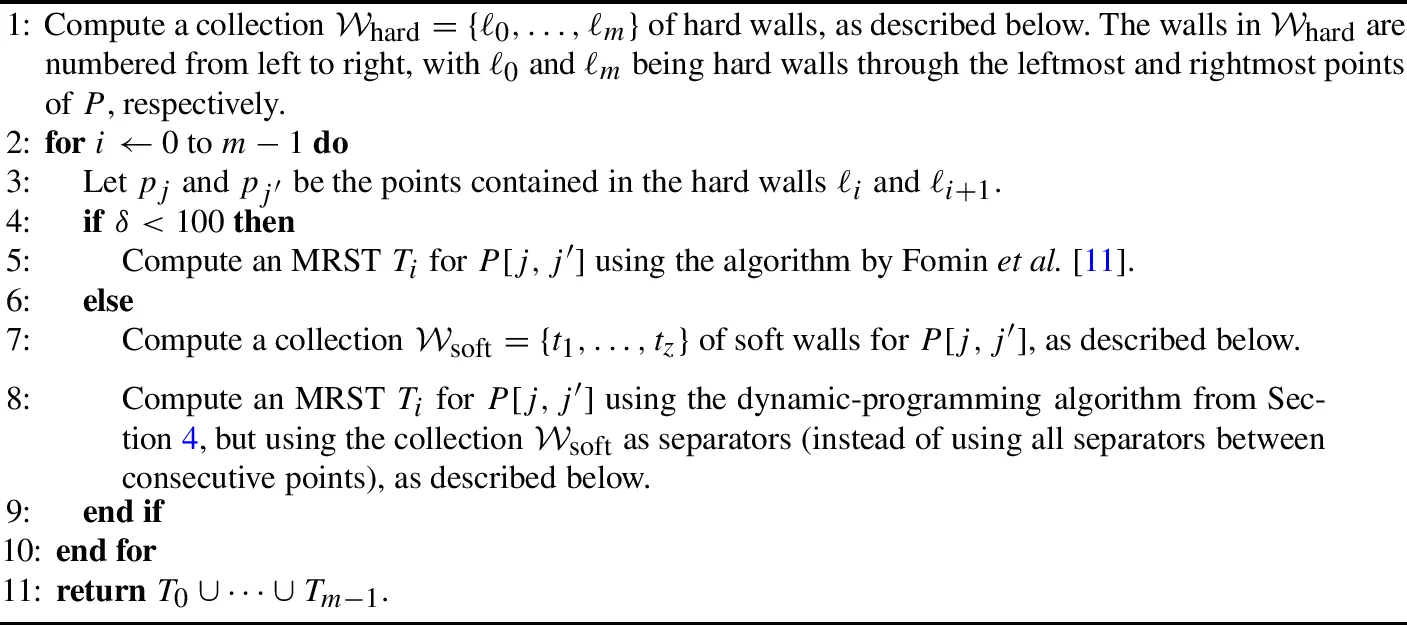
ComputeMRST(*P*)

**Computing hard walls.** Let P[i,i+4] be a subset of points from *P*, and let ℓ be the vertical line through $p_{i+2}$. We call ℓ a *hard wall* if P[i,i+4] has the following properties:

- $$\Delta _j > \delta $$ for all $$i\leqslant j\leqslant i+3$$
- $$y_{i+1}< y_{i+2} < y_{i+3}$$

**Fig. 12:**
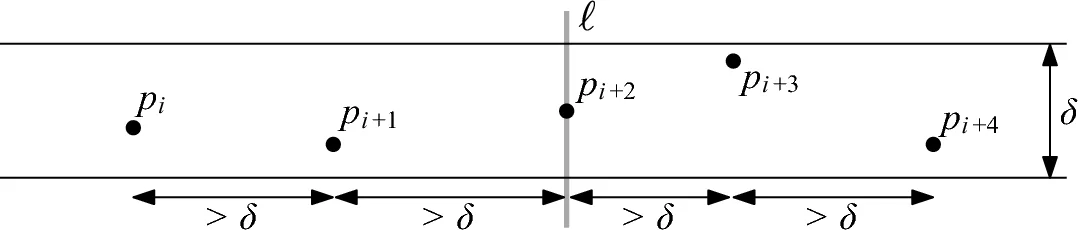
Example of a hard wall

See Figure 12 for an example of a hard wall. A hard wall indeed splits the problem into independent subproblems, as shown in the lemma below.

### Lemma 13

Let ℓ be a hard wall, defined by the subset P[i,i+4]. Let $T_1$ be an MRST on P[1,i+2] and let $T_2$ be an MRST on P[i+2,n]. Then $\Vert T\Vert = \Vert T_1\Vert + \Vert T_2\Vert$ and so $T_1 \cup T_2$ is an MRST on *P*.

### Proof

**Fig. 13:**
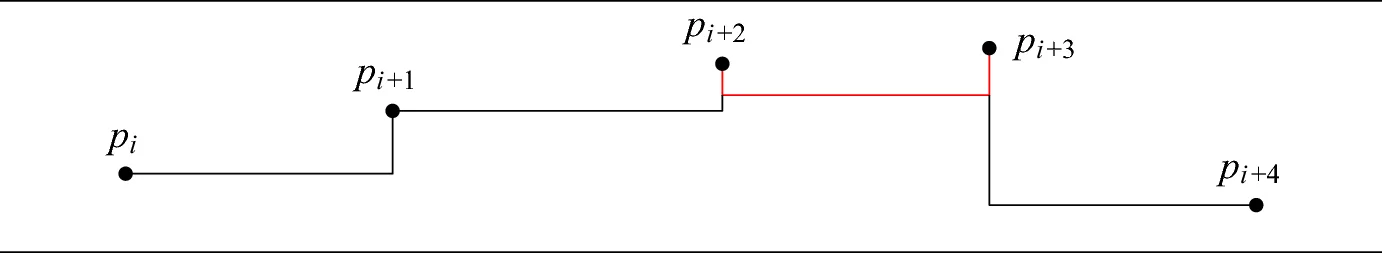
Illustration for the proof of Lemma 13 showing an MRST *T* where ${{\,\textrm{degree}\,}}_T(p_{i+2})=1$. In red, the *U*-shape showing that *T* is not a Hwang tree

Let *T* be a Hwang tree on *P*. By Observation 2 we know that an MRST on *P* is monotonic at $s_i,\ldots ,s_{i+3}$. The monotonicity at $s_{i+1}$ and $s_{i+2}$ implies that ${{\,\textrm{degree}\,}}_T(p_{i+2})\leqslant 2$. If ${{\,\textrm{degree}\,}}_T(p_{i+2})=2$ then splitting *T* at $p_{i+2}$ results in subtrees on P[1,i+2] and P[i+2,n]—this follows from the monotonicity at $s_{i+1}$ and $s_{i+2}$—and so we are done. Now assume for a contradiction that ${{\,\textrm{degree}\,}}_T(p_{i+2})=1$. Then the incident edge of $p_{i+2}$ must be vertical. For now, assume that $p_{i+2}$ is the top endpoint of this edge. Then the (single) edge of *T* crossing $s_{i+2}$ must reach the vertical line through $p_{i+3}$ at a point *q* that lies somewhere below $p_{i+3}$. The monotonicity at $s_{i+3}$ then implies that *q* must be connected to $p_{i+3}$ by a vertical segment, thus creating a U-shape and contradicting that *T* is a Hwang tree. See Figure 13 for an example.

Above we assumed that $p_{i+2}$ is the top endpoint of its incident edge. If $p_{i+2}$ is the bottom endpoint then we can apply a similar argument, this time creating a U-shape between $p_{i+2}$ and $p_{i+1}$ instead of between $p_{i+2}$ and $p_{i+3}$. □

The next lemma gives a bound on the probability that P[i,i+4] is a hard wall.

### Lemma 14

$$\mathbb {P}\big [\; P[i,i+4] \text{ defines } \text{ a } \text{ hard } \text{ wall } \;\big ] \ = \ e^{-4 \delta }/6$$ for all $$1\leqslant i\leqslant n-4$$.

### Proof

Recall that the spacings $\Delta _j$ are drawn independently from an exponential distribution with rate 1. Hence, $\mathbb {P}[\Delta _j>\delta ]=e^{-\delta }$ for all *j*. Since the spacings are independent, the probability that all four spacings between the points in P[i,i+4] are greater than δ is $e^{-4\delta }$. Finally, $\mathbb {P}[y_{i+1}<y_{i+2}<y_{i+3}]=1/6$, since all *y*-coordinates are chosen uniformly at random from [0,δ] and so all six orderings of $y_{i+1}<y_{y+2}<y_{i+3}$ are equally likely. □

The set $\mathcal {W}_{\textrm{hard}}$ of hard walls is now computed in the following straightforward manner: we check for all i:=5j+1 with $j\in \{0,\ldots ,\left\lfloor n/5 \right\rfloor -1\}$ whether P[i,i+4] defines a hard wall; if so, we add the corresponding hard wall to $\mathcal {W}_{\textrm{hard}}$. Note that this takes only *O*(*n*) time in total, as each of the *O*(*n*) candidate hard walls can be checked in *O*(1) time.

**Fig. 14:**
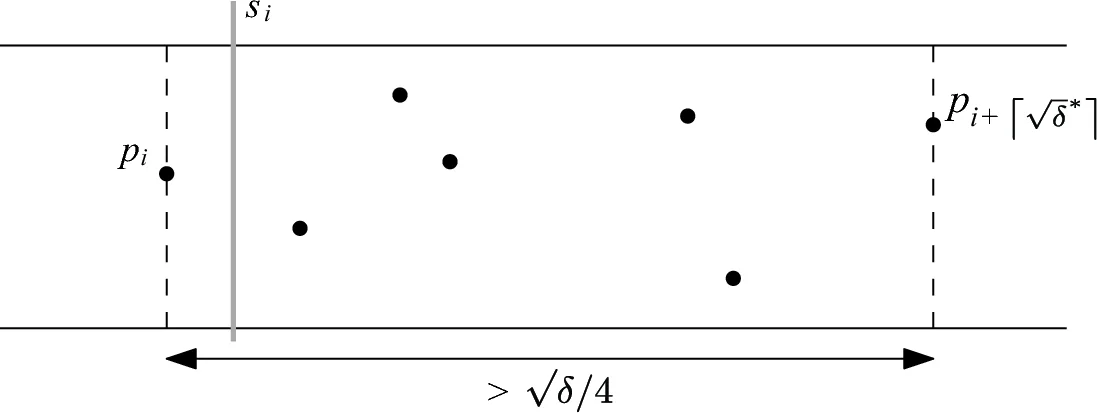
Example of a soft wall

**Computing soft walls.** Let $P[i,i+\lceil \sqrt{\delta } \rceil ]$ be a subset of $\lceil \sqrt{\delta } \rceil +1$ points from *P* such that $x_{i+\lceil \sqrt{\delta } \rceil } - x_i > \lceil \sqrt{\delta } \rceil /4$. Then we call the separator $s_i$—recall that $s_i$ is the separator between $p_i$ and $p_{i+1}$—a *soft wall*. See Figure 14 for an example.

### Lemma 15

Let $\delta \geqslant 100$. Let $s_i$ be a soft wall, defined by $P[i,i+\lceil \sqrt{\delta } \rceil ]$. Then $s_i$ is crossed $O(\sqrt{\delta })$ times by an MRST. Furthermore, even under the assumption that $\Delta _j < \delta$ for all $1 \leqslant j \leqslant n-1$, we have

14 $$\begin{aligned} \mathbb {P}\left[ \; P\left[ i,i+\big \lceil \sqrt{\delta } \big \rceil \right] \text{ defines } \text{ a } \text{ soft } \text{ wall } \; \right] \ \geqslant \ 1 - 2^{3 - \big \lceil \sqrt{\delta } \big \rceil /2} \text{ for } \text{ all } 1\leqslant i\leqslant n-\big \lceil \sqrt{\delta } \big \rceil \text{. } \end{aligned}$$

### Proof

The fact that $s_i$ is crossed at most $O(1+\lceil \sqrt{\delta } \rceil )=O(\lceil \sqrt{\delta } \rceil )$ times follows immediately from Lemma 7, by taking $c_1=0$ and $c_2=1/4$. (To be precise, $s_i$ is crossed at most $36 + 33\sqrt{\delta }$ times.) It remains to derive a lower bound on the probability that $P[i,i+\lceil \sqrt{\delta } \rceil ]$ is a soft wall, given that $\Delta _j < \delta$ for all $1 \leqslant j \leqslant n-1$. We have

$$\begin{aligned}&\mathbb {P}\left[ x_{i+\big \lceil \sqrt{\delta } \big \rceil } - x_i> \big \lceil \sqrt{\delta } \big \rceil /4 \right] \\&= 1 - \mathbb {P}\left[ \sum _{j=i}^{i+\big \lceil \sqrt{\delta } \big \rceil -1} \Delta _j \leqslant \big \lceil \sqrt{\delta } \big \rceil /4 \right] \\&\geqslant 1 - \inf _{t>0} e^{t \big \lceil \sqrt{\delta } \big \rceil /4} \left( \mathbb {E}[e^{-t\Delta _1}] \right) ^{\big \lceil \sqrt{\delta } \big \rceil -1}&\text {by the Chernoff bound}\\&= 1 - \inf _{t>0} e^{t \big \lceil \sqrt{\delta } \big \rceil /4} \left( \int _{x=0}^{\delta } e^{-tx}e^{-x} \frac{1}{1-e^{-\delta }} \,\textrm{d}x \right) ^{\big \lceil \sqrt{\delta } \big \rceil -1}&\Delta _1 \sim \textrm{Exp}(1) \text { and } \Delta _1 \leqslant \delta \\&= 1 - \inf _{t>0} e^{t \big \lceil \sqrt{\delta } \big \rceil /4} \left( \frac{e^{\delta }-e^{-\delta t}}{\left( e^{\delta }-1 \right) (t+1)} \right) ^{\big \lceil \sqrt{\delta } \big \rceil -1}\\&> 1 - \inf _{t>0} 2e^{t \big \lceil \sqrt{\delta } \big \rceil /4} \left( \frac{1}{t+1} \right) ^{\big \lceil \sqrt{\delta } \big \rceil -1}&\text {since }\delta \geqslant 100 \\&\geqslant 1 - 2e^{3\big \lceil \sqrt{\delta } \big \rceil /4} 4^{1-\big \lceil \sqrt{\delta } \big \rceil }&\text {pick } t = 3\\&> 1 - 2^{3 - \big \lceil \sqrt{\delta } \big \rceil /2} \end{aligned}$$

□

Recall that in Algorithm ComputeMRST we need to compute soft walls for every subset $P[j,j^{\prime }]$ between two consecutive hard walls (including the points on those two hard walls). To this end we check whether $$P[p_r,\ldots ,p_{r+\lceil \sqrt{\delta } \rceil }]$$ forms a soft wall for all $$r:= j+i \lceil \sqrt{\delta } \rceil $$ with $$0\leqslant i\leqslant j'-\lceil \sqrt{\delta } \rceil $$.

**The dynamic-programming algorithm between two hard walls.** Recall that $X(s_i)$ is the set of points where the Hanan grid crosses $s_i$. Let $\mathcal {X}_i$ denote the family of crossing patterns $X(s_i)$ of size at most $36 + 33 \sqrt{\delta }$. We can now define a table entry *A*[*i*] for each *soft wall* $s_i$ as follows.

*A*[*i*] := a representative set of pairs (*X*, *l*) where *l* is the minimum length of a rectilinear forest adhering to $X \in \mathcal {X}_i$ at $s_i$.

**Fig. 15:**
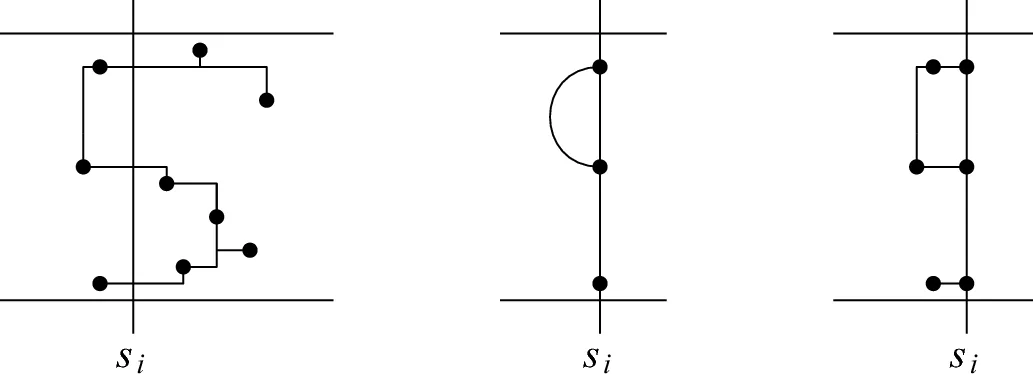
An example of an element of a table entry *A*[*i*]. On the left, an MRST *T* represented in *A*[*i*]. *T* is represented in *A*[*i*] by a pair $(X_i,l)$. In the middle, we have $X_i$. On the right, the edges contributing to the length *l*. Note that since *T* is an MRST, these edges indeed form a minimum length rectilinear forest adhering to $X_i$ at $s_i$

Here, ‘representative’ means that for every soft wall $s_i$ there exists an MRST *T* and (X,l)∈A[i] such that *T* adheres to *X* at $s_i$. We will call this *T* an MRST *represented in* *A*[*i*]. See Figure 15 for an example.

Note that *A*[*n*] contains one element, of which the length *l* equals the length of an MRST on *P*. Next we describe a recursive formula to compute the table entries. As base case, we have $A[0] = \{\{\emptyset \},0\}$.

We first give pseudocode for this part of the algorithm.

**Algorithm 2:**
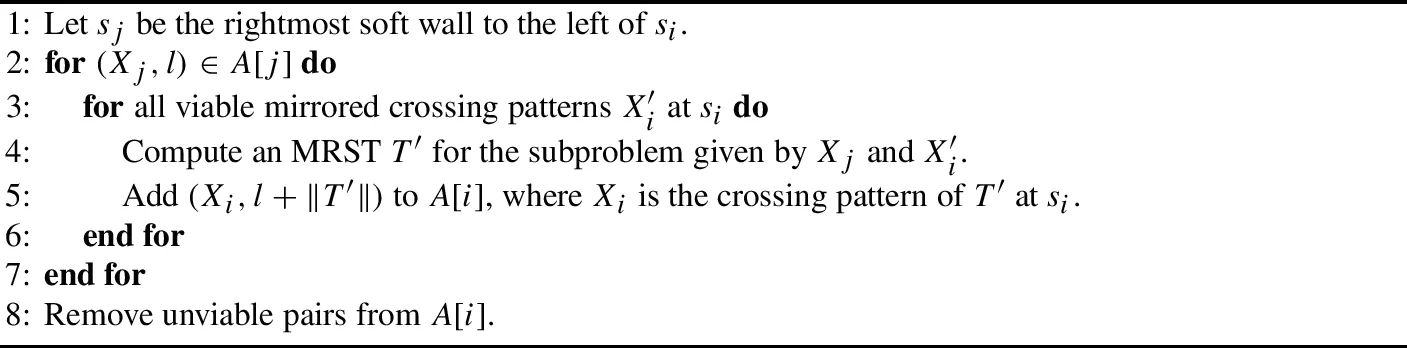
ComputeA(*i*)

Let $$s_i$$ be a soft wall, and let $$s_j$$ be the rightmost soft wall to the left of $$s_i$$. We define a mirrored crossing pattern to be a crossing pattern where the partition denotes on how the rectilinear Steiner tree is connected on the *right* hand side. Let $$(X_j,l)$$ be a pair in *A*[*j*]. Let *T* be an MRST adhering to $$X_j$$ at $$s_j$$, and adhering to some (unknown) mirrored crossing pattern $$X_i'$$ at $$s_i$$. Then there is a pair $$(X_i,l')$$ in *A*[*i*], where $l^{\prime }$ equals *l* plus the total length of the edges of *T* between $$s_j$$ and $$s_i$$. The total length of *T* between these two separators only depends on $$X_j$$ and $$X_i'$$. Let $$L(X_j,X_i')$$ denote the total length of the solution to this subproblem. Now, to compute the value of $$L(X_j, X_i')$$, we use the $$2^{O(\sqrt{n} \log n)}$$ algorithm by Fomin *et al.* [11]. Since the algorithm by Fomin *et al.* only computes Steiner trees (not forests adhering to some crossing pattern), we need to adapt our subproblem. To ensure the crossing pattern $$X_j$$, we mimic the edges on the left of $$X_j$$. For every part of $$X_j$$, we add a path of ‘virtual’ edges of length 0, connecting the points in that part. These are automatically added to the so-called shortest path RST found by the first part of the algorithm by Fomin *et al.* Since the number of virtual edges added is constant in *n*, it does not affect its running time. We ensure the crossing pattern $$X_i'$$ analogously. Given the output $T^{\prime }$ of the algorithm by Fomin *et al.*, we remove its virtual edges, and analyse its (non-mirrored) crossing pattern $$X_i$$ at $$s_i$$. We then add the pair $$(X_i, l+\Vert T'\Vert )$$ to *A*[*i*]. After doing so for all pairs in *A*[*j*] and viable mirrored crossing patterns $$X_i'$$, we may be able to remove some elements from *A*[*i*]. First, we remove any duplicates. Then, if two pairs have the same crossing pattern $$X_i$$, we need only the one with the smallest *l*.

**Fig. 16:**
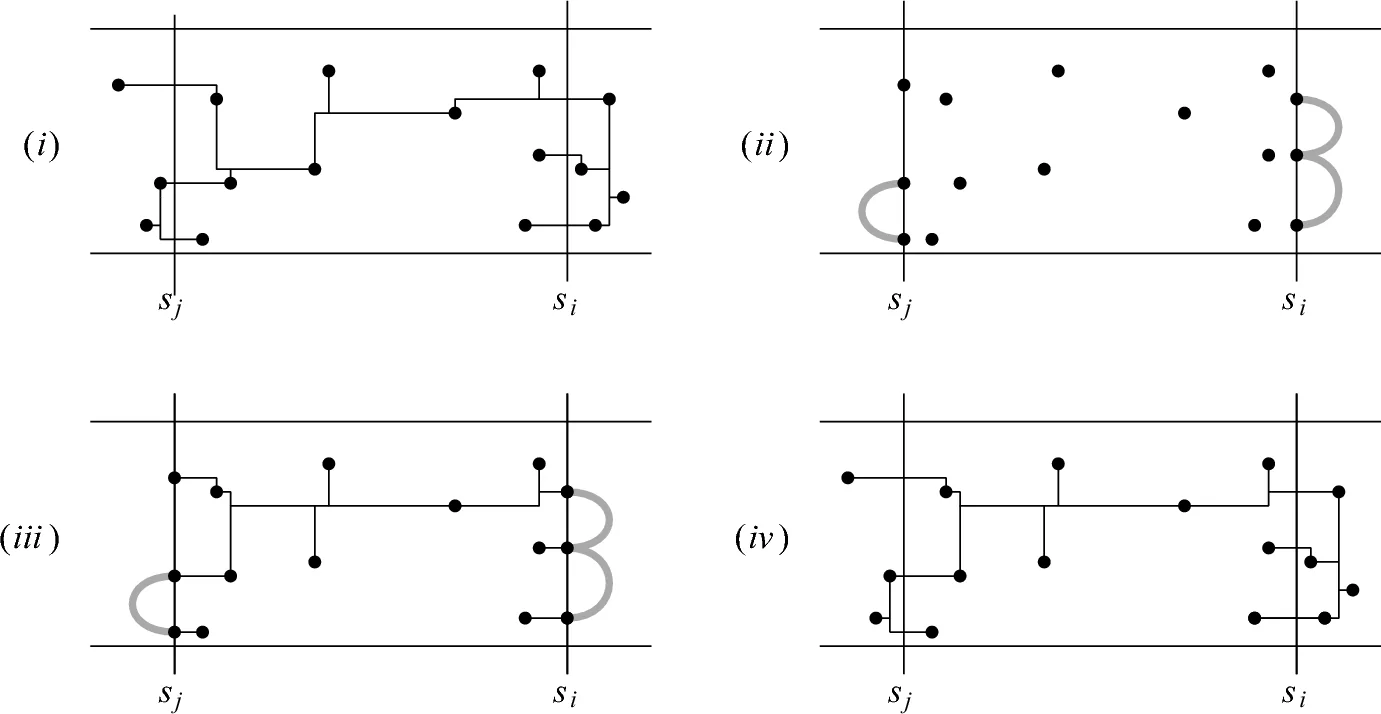
Illustration for the correctness proof of Algorithm 2. At (i), we have an MRST *T* which adheres to $$X_j$$ at $$s_j$$, and to $$X_i'$$ at $$s_i$$. At (ii), we have the corresponding subproblem. The thick grey edges denote the virtual edges of length 0. At (iii), a solution $T^{\prime }$ to the subproblem. Note that the total length of $T^{\prime }$ equals the total length of *T* between $$s_j$$ and $$s_i$$. At (iv), the new MRST $T^{\prime \prime }$ represented in *A*[*i*], obtained by combining *T* and $T^{\prime }$

We will now prove that *A*[*i*] is indeed a representative set by induction on *i*. Clearly, *A*[0] is a representative set. Now, suppose *A*[*j*] is a representative set. We will now show that after performing the above, *A*[*i*] is a representative set. See Figure 16 for an example. Since *A*[*j*] is a representative set, there exists a pair $$(X_j, l) \in A[j]$$ and an MRST *T* such that *T* adheres to $$X_j$$ at $$s_j$$. Now, *T* adheres to some mirrored crossing pattern $$X_i'$$ at $$s_i$$. Therefore, we will find an MRST $T^{\prime }$ on the subproblem defined by $$X_j$$ and $$X_i'$$, and add a pair $$(X_i,l')$$ to *A*[*i*]. Let $T^{\prime \prime }$ be the MRST on *P* obtained by exchanging the part of *T* between $$s_j$$ and $$s_i$$ for $T^{\prime }$. Note that we can do that, since *T* and $T^{\prime }$ adhere to the same crossing patterns $$X_j$$ and $$X_i'$$. Now, $T^{\prime \prime }$ is represented in *A*[*i*] by $$(X_i, l')$$.

**Analysis of the running time.** We now analyze the expected running time of Algorithm ComputeMRST. To do so, we will bound certain distributions by other distributions. To be precise, we bound the expected running time of any algorithm on a point set with a random number of points following a certain distribution by the expected running time of the algorithm on a point set with a differently distributed random number of points.

### Observation 16

([1]) Let $Y_1,Y_2$ be two discrete nonnegative random variables, such that for all k⩾0, the equation $\mathbb {P}[Y_1 \leqslant k] \geqslant \mathbb {P}[Y_2 \leqslant k]$ holds. Let *f*(*k*) be an increasing nonnegative function such that $\mathbb {E}[f(Y_2)] < \infty$. Then $\mathbb {E}[f(Y_1)] \leqslant \mathbb {E}[f(Y_2)]$.

We write $Y_1 \preccurlyeq Y_2$ to denote that for all k⩾0, the equation $\mathbb {P}[Y_1 \leqslant k] \geqslant \mathbb {P}[Y_2 \leqslant k]$ holds. We will use this to bound both the number of points between two hard walls, as well as the number of points between two soft walls.

Let $X_{1,m}$ denote the number of points between two consecutive hard walls. Here, *m* denotes the number of points to the right of the left hard wall. Now, $X_{1,m}$ is almost geometrically distributed. There are two key differences: we only check whether P[i,i+4] defines a hard wall for *i* of the form 5j+1, and the maximum number of points is bounded. Since the probability that P[i,i+4] defines a hard wall is $e^{-4\delta }/6$, we have $X_{1,m} \sim \min \{m, 1 + 5 \cdot \text {Geom}(e^{-4\delta }/6)\}$. Here, the probability mass function of $\text {Geom}(p)$ is $(1-p)^{k-1} p$. Let $X_2$ be the same distribution, but where we ignore the maximum number of points, $X_2 \sim 1 + 5 \cdot \text {Geom}(e^{-4\delta }/6)$. Then, $X_{1,m} \preccurlyeq X_2$ for all *m*.

*Analysis for the case *δ<100. We are now ready to calculate the expected running time of ComputeMRST if δ<100. We have already seen that we can find $\mathcal {W}_{\textrm{hard}}$ in *O*(*n*) time. Since $X_{1,m} \preccurlyeq X_2$ for all *m*, the expected time needed per subproblem is bounded by the time needed to run the $2^{O(\sqrt{n}\log n)}$ algorithm by Fomin *et al.* on a point set with $X_2$ points. We get:

15 $$\begin{aligned} \sum _{k=1}^\infty \left( 1-\frac{e^{-4\delta }}{6} \right) ^{k-1} \frac{e^{-4\delta }}{6} 2^{O(\sqrt{5k+1} \log (5k+1))} < \sum _{k=1}^\infty 2^{O(\sqrt{5k} \log (5k))-O(k)} = O(1)\end{aligned}$$

Since there are *O*(*n*) subproblems, this finishes the case δ<100.

*Analysis for the case *$\delta \geqslant 100$. We can use the same trick as above for the distribution of the number of points between soft walls. Here, we let $Y_{1,m}$ denote the number of points between two consecutive soft walls, given that we have found no hard walls between the hard walls defining our subproblem and where *m* is the number of points between the left soft wall and the next hard wall. We can bound $Y_{1,m}$ in three steps. First, note that the condition that no $\Delta _j$ is larger than δ is stronger than the condition that there are no hard walls between the hard walls defining our subproblem. Let $Y_{2,m}$ denote the number of points between the soft walls, given that no $\Delta _j$ is larger than δ. Then $Y_{1,m} \preccurlyeq Y_{2,m}$. Next, recall that if $\delta \geqslant 100$, by Lemma 15 the probability that $s_i$ is a soft wall is at least $1 - 2^{3 - \lceil \sqrt{\delta } \rceil /2}$, even if all $\Delta _j<\delta$. Define $Y_{3,m} \sim \min \{m, \lceil \sqrt{\delta } \rceil \cdot \text {Geom}(1 - 2^{3 - \lceil \sqrt{\delta } \rceil /2})\}$; the factor $\lceil \sqrt{\delta }\rceil$ appears because a soft wall is defined by $\big \lceil \sqrt{\delta } \big \rceil +1$ distinct points. Then $Y_{2,m} \preccurlyeq Y_{3,m}$. Finally, analogously to the hard walls, we can ignore the maximum number of points. Define $Y_4 \sim \lceil \sqrt{\delta } \rceil \cdot \text {Geom}(1 - 2^{3 - \lceil \sqrt{\delta } \rceil /2})$. We conclude that $Y_{1,m} \preccurlyeq Y_4$ for all *m*.

Let λ>0 be such that the algorithm by Fomin *et al.* runs in under $2^{\lambda \sqrt{n} \log n}$ time. For the case $\delta \geqslant 100$, the total expected time needed per subsubproblem is then bounded by

$$\begin{aligned}&\sum _{i=1}^\infty 2^{\lambda \sqrt{i \big \lceil \sqrt{\delta } \big \rceil +1}\log \left( i \big \lceil \sqrt{\delta } \big \rceil +1 \right) } \cdot \left( 2^{3-\big \lceil \sqrt{\delta } \big \rceil /2} \right) ^{i-1} \cdot \left( 1 - 2^{3-\big \lceil \sqrt{\delta } \big \rceil /2} \right) \\&< \; 2^{\big \lceil \sqrt{\delta } \big \rceil /2-3} \sum _{i=1}^\infty 2^{\lambda \sqrt{2i \big \lceil \sqrt{\delta } \big \rceil }\log \left( 2i \big \lceil \sqrt{\delta } \big \rceil \right) } \cdot 2^{(3-\big \lceil \sqrt{\delta } \big \rceil /2)i} \cdot 1\\&< \; 2^{\big \lceil \sqrt{\delta } \big \rceil } \sum _{i=1}^\infty 2^{4 \lambda (2 i\big \lceil \sqrt{\delta } \big \rceil )^{3/4}} \cdot 2^{-i \big \lceil \sqrt{\delta } \big \rceil /5} \hspace{9em} \text {since } \delta \geqslant 100&\\&< \; 2^{\big \lceil \sqrt{\delta } \big \rceil } \sum _{i=1}^\infty 2^{\big \lceil \sqrt{\delta } \big \rceil (8 \lambda i^{3/4} - i / 5)}. \end{aligned}$$

Now, for a sufficiently large *M*, we have $8 \lambda i^{3/4} - i / 5 < -i/10$ for all i⩾M. We get:

$$\begin{aligned}&2^{\big \lceil \sqrt{\delta } \big \rceil } \sum _{i=1}^\infty 2^{\big \lceil \sqrt{\delta } \big \rceil (8 \lambda i^{3/4} - i / 5)} \\&= \; 2^{\big \lceil \sqrt{\delta } \big \rceil } \left( \sum _{i=1}^{M} 2^{\big \lceil \sqrt{\delta } \big \rceil (8 \lambda i^{3/4} - i / 5)} + \sum _{i=M+1}^\infty 2^{\big \lceil \sqrt{\delta } \big \rceil (8 \lambda i^{3/4} - i / 5)} \right) \\&< \; 2^{\big \lceil \sqrt{\delta } \big \rceil } \left( M \cdot 2^{\big \lceil \sqrt{\delta } \big \rceil \max _{i \geqslant 1} (8 \lambda i^{3/4} - i / 5)} + 2^{\big \lceil \sqrt{\delta } \big \rceil } \cdot \sum _{i=M+1}^\infty 2^{- i / 10} \right) \\&= \; 2^{O\left( \sqrt{\delta } \right) }. \end{aligned}$$

Let *m* be the number of points in the corresponding subproblem defined by two hard walls. Note that the above bound is independent of *m*. Analogously to the original sparse point-set algorithm, there are $m^{O(\big \lceil \sqrt{\delta } \big \rceil )}$ possible crossing patterns per separator. In total, this algorithm therefore takes

$$ m \, \cdot m^{O\left( \sqrt{\delta } \right) } \cdot 2^{O\left( \sqrt{\delta } \right) } = m^{O\left( \sqrt{\delta } \right) } $$

expected time between two hard walls, where *m* is the number of points between those walls.

Now we are ready to compute the expected total running time of our algorithm for the case where $\delta \geqslant 100$. Clearly, the expected running time is bounded by $n^{O(\sqrt{\delta })}$, since splitting up the problem using the hard walls can only speed up the algorithm. We now prove that $2^{O\left( \delta \sqrt{\delta } \right) } n$ is an upper bound on the expected running time as well.

Let $\lambda \geqslant 1$ be a constant such that the expected running time of the algorithm on a subproblem (that is, between two hard walls) is $m^{\lambda \sqrt{\delta }}$, where *m* is the number of points of the subproblem. Let $Y_1$ be the distribution of the number of points of a subproblem. Recall that $Y_1 \preccurlyeq X_2$, where $X_2 \sim 1 + 5 \cdot \text {Geom}(e^{-4\delta }/6)$. It will be convenient to define a constant μ>1 such that $e^{-4\delta }/6 = 2^{-\mu \delta }$, so that

$$ Y_1 \preccurlyeq 1 + 5 \cdot \text {Geom}(2^{-\mu \delta }). $$

Then the total expected time needed per subproblem is:

$$\begin{aligned} \mathbb {E}\left[ Y_1^{\lambda \sqrt{\delta }} \right]&\leqslant \sum _{k=1}^\infty (1-2^{-\mu \delta })^{k-1} \cdot 2^{-\mu \delta } \cdot \left( 5k+1 \right) ^{\lambda \sqrt{\delta }} \\  &< O(1) + 2^{-\mu \delta } \sum _{k=2}^\infty 2^{4\lambda \sqrt{\delta }\log k -2^{-\mu \delta } k} .\end{aligned}$$

We split the sum into two parts, namely for k<M and k⩾M, where $M=64 \lambda ^2 \delta 2^{2 \mu \delta }$. Observe that for k⩾M we have

$$ 4\lambda \sqrt{\delta }\log k < 4\lambda \sqrt{\delta k} \leqslant \tfrac{1}{2} \cdot 2^{-\mu \delta } k. $$

Thus, we get

$$\begin{aligned}&2^{-\mu \delta } \sum _{k=2}^\infty 2^{4\lambda \sqrt{\delta }\log k -2^{-\mu \delta } k}\\&< 2^{-\mu \delta } \left( \sum _{k=2}^{M-1} 2^{4\lambda \sqrt{\delta }\log k -2^{-\mu \delta } k} + \sum _{k=M}^\infty 2^{-2^{-\mu \delta } k/2} \right) \\&< 2^{-\mu \delta } M 2^{\max _{k} \left( 4\lambda \sqrt{\delta }\log k -2^{-\mu \delta } k \right) } + \frac{2^{-\mu \delta }}{1-2^{-2^{-\mu \delta -1}}} \\&< 2^{-\mu \delta } M 2^{4\lambda \sqrt{\delta } \log M} + O(1) = 2^{O(\delta \sqrt{\delta })}, \end{aligned}$$

since $M=2^{O(\delta )}$, and λ and μ are constants, and $\frac{2^{-\mu \delta }}{1-2^{-2^{-\mu \delta -1}}} < 2 / \ln 2$. Because the total number of subproblems is obviously *O*(*n*), this brings the total expected running time to $O(n) \cdot (O(1) + 2^{O(\delta \sqrt{\delta })}) = 2^{O(\delta \sqrt{\delta })} n$.

All in all, ComputeMRST runs in *O*(*n*) expected time if δ<100, and it runs in $\min \{n^{O(\sqrt{\delta })},2^{O(\delta \sqrt{\delta })} n\}$ expected time if $\delta \geqslant 100$.

*Putting it all together.* Now all that remains is to show that the requirements for Lemma 12 hold, where we take $\mathcal {A}$ to be the algorithm described above. The only nontrivial requirement is that $T_\mathcal {A}(P_\lambda ) \leqslant T_\mathcal {A}(P)$ for all point sets *P* and *x*-axis scaling factors λ>1. Intuitively, we would expect this to hold: every wall retains its properties when scaled with a factor larger than 1, and whether a point set is checked for being a hard or soft wall solely depends on the value of δ. Therefore, every wall found in *P* will also be found in $P_\lambda$. Furthermore, $P_\lambda$ could contain more walls, which should only help decrease the running time.

Unfortunately, it does not necessarily hold, since we have only proven bounds on the running times, not the actual running times themselves. However, it is easy to show the existence of an algorithm $\mathcal {B}$ of which we know the exact expected running time of every step. To prove this, we will use the same method as used in [1].

Algorithm $\mathcal {B}$ is simply algorithm $\mathcal {A}$, but after every step it waits as long as necessary to make its running time for that step equal to the bound calculated for this step. So, for example, if we have previously calculated a bound of $5 n^2$ time for some step, and $\mathcal {A}$ actually takes 2+3n time for some instance, $\mathcal {B}$ should wait for $5n^2-(2+3n)$ time afterwards.

Since we know the exact running time of $\mathcal {B}$, we get using the arguments given earlier that $T_\mathcal {B}(P_\lambda ) \leqslant T_\mathcal {B}(P)$ for all point sets *P* and *x*-axis scaling factors λ>1. Therefore, we can apply Lemma 12 on $\mathcal {B}$, giving us that the expected running time of $\mathcal {B}$ on $X_{\delta ,n}$ is *O*(*n*) if δ<100, and $\min \{n^{O(\sqrt{\delta })},2^{O(\delta \sqrt{\delta })} n\}$ if $\delta \geqslant 100$. Since $\mathcal {B}$ is slower than $\mathcal {A}$, we conclude that the expected running time of $\mathcal {A}$ must have the same bound. This leads to the following lemma.

### Lemma 17

Let *P* be a set of *n* points generated randomly inside a rectangle of height δ and expected width *n*, generated according to the procedure described earlier. Then an MRST on *P* can be found in $\min \{n^{O(\sqrt{\delta })},2^{O(\delta \sqrt{\delta })} n\}$ expected time.

The next theorem follows immediately from Lemma 17 by observing that a point set *P* generated uniformly at random can be sorted on *x*-coordinate in *O*(*n*) expected time using BucketSort [8].

### Theorem 18

Let *P* be a set of *n* points generated independently and uniformly at random inside the rectangle $[0,n]\times [0,\delta ]$. Then an MRST on *P* can be found in $\min \{n^{O(\sqrt{\delta })},2^{O(\delta \sqrt{\delta })} n\}$ expected time.

## Concluding remarks

Our paper contains two main results on Minimum Rectilinear Steiner Tree. First, we proved that for sparse point sets in a strip of width δ, an MRST can be found in $n^{O(\sqrt{\delta })}$ time. Second, we gave a $\min \{n^{O(\sqrt{\delta })},2^{O(\delta \sqrt{\delta })} n\}$ expected running time algorithm for random point sets. For $\delta =\Theta (n)$ the running time equals the $2^{O(\sqrt{n} \log n)}$ of the algorithm for arbitrary point sets in the plane [11]. A challenging open problem is to see if an algorithm with running time $2^{O(\sqrt{\delta }\log \delta )}\text{ poly }(n)$ is possible. Another direction for future research is to study the problem in higher dimensions. We believe that our algorithmic results may carry over to $\mathbb {R}^d$ to points that are almost collinear, that is, that lie in a narrow cylinder. Generalizing the results to, say, points lying in a narrow slab will most likely be more challenging.

More generally, we believe that it is interesting to study the parameterized complexity of geometric problems using a “geometric parameter”. For problems involving planar point sets, the strip width δ is a natural parameter, which is interesting because it explores the boundary between the 1-dimensional and 2-dimensional version of the problem. We have studied this for TSP in a previous paper [1] and for Minimum Rectilinear Steiner Tree in the current paper, but many other problems can be studied from this perspective as well.

## Data Availability

Data sharing not applicable to this article as no datasets were generated or analysed during the current study.

## Footnotes

1 When we refer to the “interior” of a segment, we always mean its relative interior, i.e. the segment excluding its endpoints.

## References

[1] Alkema, H., de Berg, M., van der Hofstad, R., Kisfaludi-Bak, S.: Euclidean TSP in narrow strips. Discret. Comput. Geom. **71**(4), 1456–1506 (2024). 10.1007/S00454-023-00609-7

[2] Althaus, E.: Berechnung optimaler Steinerbäume in der ebene. Master’s thesis, Max-Planck-Institut für Informatik in Saarbrücken, Universität des Saarlandes (1998)

[3] Björklund, A., Husfeldt, T., Kaski, P., Koivisto, M.: Fourier meets Möbius: fast subset convolution. In: Proc. 39th Annual ACM Symposium on Theory of Computing (STOC 2007), pp. 67–74. ACM, New York, USA (2007). 10.1145/1250790.1250801

[4] Brazil, M., Zachariasen, M.: Optimal Interconnection Trees in the Plane: Theory, Algorithms, and Applications, Springer, Switzerland (2015). 10.1007/978-3-319-13915-9

[5] Brazil, M., Zachariasen, M.: Steiner trees for fixed orientation metrics. J. Glob. Optim. **43**(1), 141–169 (2009). 10.1007/s10898-008-9305-y

[6] Brazil, M., Zachariasen, M.: The uniform orientation Steiner tree problem is NP-hard. Int. J. Comput. Geom. Appl. **24**(2), 87–106 (2014). 10.1142/S0218195914500046

[7] Brazil, M., Thomas, D.A., Weng, J.F., Zachariasen, M.: Canonical forms and algorithms for Steiner trees in uniform orientation metrics. Algorithmica **44**(4), 281–300 (2006). 10.1007/s00453-005-1178-6

[8] Cormen, T.H., Leiserson, C.E., Rivest, R.L., Stein, C.: Introduction to Algorithms, 3rd edn. MIT Press and McGraw-Hill, Cambridge, USA (2009)

[9] Deneen, L., Shute, G., Thomborson, C.: A probably fast, provably optimal algorithm for rectilinear Steiner trees. Random Structures & Algorithms **5**, 535–557 (1994). 10.1002/rsa.3240050405

[10] Dreyfus, S.E., Wagner, R.A.: The Steiner problem in graphs. Networks **1**(3), 195–207 (1971). 10.1002/net.3230010302

[11] Fomin, F., Lokshtanov, D., Kolay, S., Panolan, F., Saurabh, S.: Subexponential algorithms for rectilinear Steiner tree and arborescence problems. ACM Trans. Algorithms **16**, 1–37 (2020). 10.1145/3381420

[12] Garey, M.R., Johnson, D.S.: The rectilinear Steiner tree problem is NP-complete. SIAM J. Appl. Math. **32**(4), 826–834 (1977)

[13] Garey, M.R., Graham, R.L., Johnson, D.S.: The complexity of computing Steiner minimal trees. SIAM J. Appl. Math. **32**(4), 835–859 (1977)

[14] Gilbert, E.N., Pollak, H.O.: Steiner minimal trees. SIAM J. Appl. Math. **16**(1), 1–29 (1968)

[15] Hanan, M.: On Steiner’s problem with rectilinear distance. SIAM J. Appl. Math. **14**(2), 255–265 (1966)

[16] Hwang, F.K.: On Steiner minimal trees with rectilinear distance. SIAM J. Appl. Math. **30**(1), 104–114 (1976)

[17] Juhl, D., Warme, D., Winter, P., Zachariasen, M.: The geoSteiner software package for computing Steiner trees in the plane: an updated computational study. Math. Program. Comput. **10**, 487–532 (2018). 10.1007/s12532-018-0135-8

[18] Nederlof, J.: Fast polynomial-space algorithms using inclusion-exclusion. Algorithmica **65**(4), 868–884 (2013). 10.1007/s00453-012-9630-x

[19] Thomborson, C.D., Deneen, L.L., Shute, G.M.: Computing a rectilinear Steiner minimal tree in time. In: Proc. International Workshop on Parallel Algorithms and Architectures. Lecture Notes in Computer Science, vol. 269, pp. 176–183 (1987). 10.1007/3-540-18099-0_44

[20] Thomborson, C.D., Alpern, B., Carter, L.: Rectilinear Steiner tree minimization on a workstation. In: Proc. DIMACS Workshop on Computational Support for Discrete Mathematics. DIMACS Series in Discrete Mathematics and Theoretical Computer Science, vol. 15, pp. 119–136 (1992). 10.1090/dimacs/015/10

[21] Warme, D.: Spanning trees in hypergraphs with applications to Steiner trees. PhD thesis, University of Virginia (1998)

[22] Widmayer, P., Wu, Y., Wong, C.K.: On some distance problems in fixed orientations. SIAM J. Comput. **16**(4), 728–746 (1987). 10.1137/0216049

[23] Winter, P.: An algorithm for the Steiner problem in the Euclidean plane. Networks **15**(3), 323–345 (1985). 10.1002/net.3230150305

